# Generative Distribution Prediction: A Unified Approach to Multimodal Learning

**DOI:** 10.1007/s10994-026-07148-1

**Published:** 2026-08-31

**Authors:** Xinyu Tian, Xiaotong Shen

**Affiliations:** School of Statistics, University of Minnesota, Minneapolis, MN 55455 USA

**Keywords:** Generative models, Prediction, Multimodality, Transfer learning, Unstructured data

## Abstract

Accurate prediction for multimodal data—including tabular, textual, and visual inputs or outputs—is essential for advancing analytics across diverse application domains. Existing methods often struggle to integrate heterogeneous data types while maintaining strong predictive performance. We introduce Generative Distribution Prediction (GDP), a model-agnostic framework that leverages high-fidelity multimodal synthetic data generated from the conditional distribution of interest, such as via conditional diffusion models, to enhance prediction across both structured and unstructured modalities. GDP is compatible with any expressive generative model and naturally supports transfer learning for domain adaptation. We provide a rigorous theoretical foundation for GDP, establishing statistical guarantees on its predictive accuracy when diffusion models serve as the generative backbone. By estimating the underlying data-generating distribution and enabling loss-adapted risk minimization, GDP delivers accurate point predictions in broad multimodal settings. We empirically validate GDP on a range of supervised learning tasks, including adaptive quantile regression, modal regression, tabular prediction, image captioning, and question answering, demonstrating its versatility and effectiveness across domains.

## Introduction

The advent of big data and the proliferation of diverse data sources have catalyzed a growing interest in multimodal learning. Multimodal data combines information from multiple sources or modalities. Each modality offers unique insights into the underlying phenomena, and integrating these heterogeneous data types holds the potential for more comprehensive models and enhanced predictive performance (Baltrusaitis et al., 2018; Ngiam et al., 2011).

In contexts such as healthcare, integrating medical records, imaging, and genomics has shown potential in enhancing outcomes (Esteva et al., 2017). For applications like autonomous driving, combining data from cameras, lidar, and radar promotes superior decision-making (Chen et al., 2017). Additionally, in domains such as social media analysis and emotion recognition, merging text, images, and physiological signals offers substantial benefits (Cowie & Cornelius, 2003; Wang et al., 2015). These applications underscore the necessity for models capable of effectively processing multimodal data. However, integrating heterogeneous modalities poses significant challenges due to variations in structural characteristics, representation, and statistical properties (Baltrusaitis et al., 2018; Ramachandram & Taylor, 2017). For instance, images are characterized by high dimensionality, text is inherently sequential, and tabular data is structured, which complicates the fusion process.

**Knowledge gap.** Traditional methods often utilize prediction models designed for single-modal or tabular data. The complexity of unstructured data, including challenges like high dimensionality and the absence of natural structure in embeddings, further obstructs multimodal learning (Dai et al., 2022). Most contemporary multimodal pipelines rely on modality-specific supervised blocks that ultimately deliver only point predictions—conditional means, medians, or quantiles. Consequently, they (i) discard distributional shape and calibrated uncertainty, (ii) under-utilize dependencies that arise only at the joint level, and (iii) lack a common distributional decision principle that can adapt to varied user-specified prediction targets.

To address these challenges, we introduce *Generative Distribution Prediction* (GDP). This framework integrates multimodal data by generating *conditional synthetic samples* from a generative model trained to approximate the conditional distribution of interest. GDP builds on the key insight that generative models—including diffusion models (Ho et al., 2020; Kim et al., 2022; Kotelnikov et al., 2023; Lin et al., 2024; Sohl-Dickstein et al., 2015; Yuan et al., 2023; Zhang et al., 2023; Zheng, 2022) and normalizing flows (Kingma & Dhariwal, 2018)—can capture rich structural dependencies that supervised learning methods often overlook, as the latter typically focus on limited distributional summaries such as conditional means or quantiles. The central premise of this work is that, when the conditional generator is sufficiently accurate, modeling the underlying data-generating mechanism can improve the generalizability of supervised predictors and enable flexible loss-adapted prediction.

An additional advantage of the GDP framework is its compatibility with transfer learning: the target generative model can be fine-tuned from a pretrained source model, with embedding dimensions adapted to improve generation fidelity. This strategy leverages the broad representational knowledge encoded in large-scale models trained on extensive datasets, while tailoring it to the specific demands of multimodal integration, thereby further improving performance on downstream tasks.

GDP is closely related to, but distinct from, established distribution-aware prediction paradigms. Like probabilistic forecasting (Gneiting & Katzfuss, 2014) and Bayesian decision theory (Berger, 1985), GDP separates distributional modeling from the downstream decision rule. Unlike many task-specific probabilistic predictors, however, GDP uses conditional generation to approximate the response distribution and then reuses the same generated distribution under different losses, such as squared, quantile, modal, or semantic dissimilarity losses. Thus, GDP should be interpreted as a unified distributional principle rather than a single universal architecture: across modalities, the encoders, loss functions, and generative backbones may differ.

GDP, a distribution-centric approach, unlocks three key advantages, each supported in the paper by both theory and numerical experiments:

- Higher predictive accuracy for distributional characteristics. By learning the entire conditional distribution rather than a single summary (mean or mode), GDP can directly optimize any functional of interest—means, medians, quantiles, and tail risks, and does so jointly. Theoretical error bounds and extensive experiments support that this distribution-centric view can deliver lower point-forecast errors and better calibration than point-estimation or single-modal baselines in the studied settings.
- Multimodal learning. GDP treats heterogeneous signals, text, images, and tabular data through a common distribution-learning and risk-minimization principle. Across applications, GDP may employ modality-appropriate encoders, task-specific losses, and different generative backbones, while retaining a common distributional decision rule.
- Generative capability. GDP based on a generative model can draw plausible future scenarios through data augmentation and support risk-aware or simulation-based decision-making while remaining consistent with observed data.

The contributions of this article are as follows:

1. Generative Distribution Prediction for multimodal supervised learning: This paper introduces the Generative Distribution Prediction framework for multimodal supervised learning. GDP uses conditional generative models, including diffusion models and large language-image models, to approximate response distributions and then forms predictions through loss-adapted risk minimization. This distributional formulation distinguishes GDP from direct point-prediction methods and supports tasks such as quantile regression, modal regression, image captioning, and question answering. As shown in Sect. 4, GDP performs competitively or improves over the evaluated baselines in the studied settings, with gains depending on the task, modality, and fitted generator.
2. Theoretical foundations: We establish theoretical foundations for diffusion-based GDP. The analysis identifies two performance factors: (1) generation error, measuring the discrepancy between the fitted synthetic distribution and the target data-generating distribution, and (2) synthetic sampling error, which decreases with the inference-time sample size *m*. This result explains why larger *m* can reduce finite-sampling variation while leaving a generator-dependent error term, and it provides the basis for practical validation-based choices of *m* and generator adequacy checks.
3. Advancements in domain adaptation: We develop a domain adaptation strategy within the GDP framework using a dual-level embedding mechanism to bridge source and target domains. This embedding structure is designed to reduce distributional mismatch while preserving information useful for prediction. The experiments show improved performance in the studied shifted-domain settings, suggesting that conditional generation combined with transfer learning can be useful when source and target tasks share distributional structure.

The rest of the paper is structured as follows: Sect. 2 elaborates on the GDP framework for multimodal supervised learning and presents theoretical error bounds for the accuracy of point predictions. Section 3 explores multimodal learning and domain adaptation using dual-level shared embeddings, facilitating transfer learning between source and target tasks. Building on this foundation, we develop multimodal diffusion models to illustrate the benefits of transfer learning. Section 4 compares GDP with standard and task-specific supervised baselines across tabular prediction with domain adaptation, question answering, image captioning, adaptive quantile regression, and modal regression. Finally, Sect. 5 discusses the practical scope and limitations of the method.

## Generative Distribution Prediction

In multimodal supervised learning, the objective is to predict the outcomes of response variables (outputs) based on predictors (inputs) for new, unseen data by learning the underlying relationships from paired input–output data. A key challenge in this framework is domain adaptation, where a supervised model trained on the source domain must adapt to perform well in a different but related target domain. For example, a credit scoring model trained on data from a high-risk population adapts to a low-risk population where defaults are rare. This scenario, known as response shift, occurs when the conditional relationship between input features (covariates) and the response variable remains consistent across domains. Still, the distribution of the response variable differs between the source and target domains. Similarly, covariate shift arises when the marginal distribution of the covariates changes between domains, while the conditional distribution of the response given the covariates remains unchanged (Sugiyama et al., 2007). Domain adaptation also encompasses the standard case of supervised learning when the data distribution is consistent across both domains.

To predict the outcomes of response variables $\boldsymbol{Y}_t$ given a new predictor value $\boldsymbol{X}_t=\boldsymbol{x}_t^{\text {new}}$, we consider the predictive probability $P_{\boldsymbol{y}_t|\boldsymbol{x}_t^{\text {new}}}$, the conditional probability of $\boldsymbol{Y}_t$ given $\boldsymbol{X}_t=\boldsymbol{x}_t^{\text {new}}$, with subscript *t* denoting the target task. Here $\boldsymbol{Y}_t$ and $\boldsymbol{X}_t$ can be tabular, unstructured, such as text, or both. This probability, supported by decision theory (Berger, 1985), captures the direct relationship between $\boldsymbol{X}_t$ and $\boldsymbol{Y}_t$, ensuring accurate predictions by accounting for how different values of $\boldsymbol{X}_t$ affect the likelihood of $\boldsymbol{Y}_t$.

### Generative Distribution Prediction

Generative Distribution Prediction generates predictive distributions and corresponding point predictions by synthesizing data to replicate the conditional distribution of the response given the predictors. Although GDP is capable of providing uncertainty measures from these predictive distributions, this article focuses exclusively on point prediction. The GDP methodology involves two key steps:

**Step 1: Constructing a conditional generator for domain adaptation.** Using transfer learning, we fine-tune a target generative model based on a pre-trained source model and the target training sample $D = \{(\boldsymbol{x}_t^i, \boldsymbol{y}_t^i)\}_{i=1}^{n}$, leveraging dual-level shared embeddings (DSE) as discussed in Sect. 3.1. In scenarios where transfer learning is not applicable, the conditional generator may be trained directly on *D*.

**Step 2: Using synthetic data for point prediction.** The conditional generator produces a synthetic sample $D_{\boldsymbol{x}_t^{\text {new}}} = \{(\tilde{\boldsymbol{y}}_t^k, \boldsymbol{x}_t^{\text {new}})\}_{k=1}^m$, representing the responses $\tilde{\boldsymbol{Y}}_t\sim \hat{P}_{{\boldsymbol{y}}_t | \boldsymbol{x}_t}(\tilde{\boldsymbol{y}}_t |\boldsymbol{x}_t^{\text {new}})$ corresponding to a given predictor value $\boldsymbol{x}_t^{\text {new}}$, where $\hat{P}_{{\boldsymbol{y}}_t | \boldsymbol{x}_t}$ represents the generation distribution obtained in Step 1. To obtain a point prediction, we first construct an empirical prediction loss $m^{-1} \sum _{k=1}^m \ell (\theta (\boldsymbol{x}_t^{\text {new}}),\tilde{\boldsymbol{y}}_t^k)$, where the loss function *ℓ * (for example, the squared loss) evaluates the prediction performance between the synthetic responses $\{\tilde{\boldsymbol{y}}_t^k\}$ by its prediction rule $\{\theta (\boldsymbol{x}_t^{\text {new}})\}$. The point prediction is then determined by minimizing this loss:

1 $$\begin{aligned} \hat{\boldsymbol{\theta }}(\boldsymbol{x}_t^{\text {new}}) = {\mathop {{\mathrm{arg\, min}}}\limits _{\boldsymbol{\theta }(\boldsymbol{x}_t^{\text {new}}) \sim \Theta (\boldsymbol{x}_t^{\text {new}})}} m^{-1} \sum _{k=1}^m \ell (\boldsymbol{\theta }(\boldsymbol{x}_t^{\text {new}}),\tilde{\boldsymbol{y}}_t^k). \end{aligned}$$

This framework encompasses multiple tasks beyond simple point estimation:

- Point estimation: By choosing $\Theta (\boldsymbol{x}_t^{\textrm{new}})$ to be the support of the empirical conditional distribution $\hat{P}_{\boldsymbol{y}_t\mid \boldsymbol{x}_t}(\tilde{\boldsymbol{y}}_t \mid \boldsymbol{x}_t^{\textrm{new}})$, and selecting *ℓ * as the squared loss, quantile loss, or a kernel-based loss, one recovers mean regression, quantile regression (Koenker & Bassett, 1978), or modal regression (Liu et al., 2023), respectively.
- Density estimation: If $\Theta (\boldsymbol{x}_t^{\textrm{new}})$ is set to the class of conditional density functions and $\ell (\boldsymbol{\theta },y)=-\log \boldsymbol{\theta }(y)$, then $\hat{\boldsymbol{\theta }}$ becomes the conditional maximum-likelihood density estimator (Cule et al., 2010).
- Selection inference: When $\Theta (\boldsymbol{x}_t^{\textrm{new}})$ consists of a finite set of generated samples, this formulation specializes to selection-inference over the outputs of the generative model (Creswell et al., 2023).

This decision rule is related to minimum Bayes risk (MBR) decoding (Eikema, 2022; Kumar & Byrne, 2004) and to self-consistency in LLM reasoning (Wang et al., 2023), since all use multiple model outputs to improve a final decision. GDP differs in two main respects. First, GDP is formulated for supervised learning: it learns or approximates the conditional distribution of a response given predictors and then applies a user-specified loss to obtain a task-specific estimator. Second, the estimator space $\Theta (\boldsymbol{x}_t^{\textrm{new}})$ need not be the finite set of generated candidates. It may be continuous, structured, or a class of functions, so the mean, quantile, mode, density, or a generated candidate can all be recovered by choosing different losses and estimator spaces. In particular, when GDP restricts the estimator space to the generated candidate set and minimizes the corresponding expected loss over that set, standard MBR decoding is included as a special case. Thus, MBR decoding is one important instance of GDP rather than the full framework. Likewise, self-consistency in LLMs can be viewed as a particular consensus-based decision rule on sampled outputs, whereas GDP is not limited to majority-vote or consistency-based selection and instead permits arbitrary user-specified losses and estimator spaces.

This methodology offers a common distributional prediction principle for both tabular and unstructured data. Section 2.2 provides a statistical guarantee for diffusion-based GDP, while Sect. 4 evaluates the resulting prediction framework and compares it with classical prediction methods that minimize (1) using the original sample *D*.

### Theory: GDP’s Predictive Risk

This subsection establishes a theoretical foundation linking generation error in synthetic data replication—or estimation error in the global data distribution—to local point predictions within the GDP methodology. It highlights the essential role of precise distribution estimation, achieved through high-fidelity data generation, in minimizing downstream risk within the GDP framework. By ensuring accurate synthetic data generation, the GDP approach facilitates robust predictions across diverse loss functions through risk minimization, as defined in (1). In essence, effectively estimating the data-generating distribution enhances performance across downstream prediction tasks. For instance, high-fidelity synthetic data replication leads to strong predictive performance under various loss functions, including absolute loss, hinge loss, and squared error loss, demonstrating the flexibility and effectiveness of the proposed approach.

To formalize this, the excessive risk $R(\boldsymbol{\theta }_0, \boldsymbol{\theta })$ quantifies the risk associated with loss *ℓ * in (1):

2 $$\begin{aligned} R(\boldsymbol{\theta }_0(\boldsymbol{x}_t), \boldsymbol{\theta }(\boldsymbol{x}_t)) = \operatorname {E}_{\boldsymbol{y}_t|\boldsymbol{x}_t} \left[ \ell (\boldsymbol{\theta }(\boldsymbol{x}_t),\boldsymbol{Y}_t) - \ell (\boldsymbol{\theta }_0(\boldsymbol{x}_t),\boldsymbol{Y}_t) \right] , \end{aligned}$$

where $\operatorname {E}_{\boldsymbol{y}_t|\boldsymbol{x}_t}$ is taken concerning the true conditional distribution of $\boldsymbol{Y}_t$ given $\boldsymbol{x}_t$. The Wasserstein-1 distance is used as a metric to assess the accuracy of global distribution estimation, defined as:

3 $$\begin{aligned} W(P, Q) = \inf _{\gamma \in \Gamma (P, Q)} \int \Vert x - y\Vert \, d\gamma (x, y), \end{aligned}$$

where $\Vert \cdot \Vert$ is the Euclidean distance, and *Γ (P, Q)* is the set of all joint distributions (couplings) with marginals *P* and *Q*.

We introduce the following regularity conditions on the loss function *ℓ * to connect global distribution estimation accuracy with local point predictions:

#### Assumption 1

*(Lipschitz over bounded domain)* The loss *ℓ * satisfies the Lipschitz condition:

4 $$\begin{aligned} \sup _{(\boldsymbol{x}_t, \boldsymbol{y}_t)} |\ell (\boldsymbol{\theta }_1(\boldsymbol{x}_t), \boldsymbol{y}_t) - \ell (\boldsymbol{\theta }_2(\boldsymbol{x}_t), \boldsymbol{y}_t)| \le \beta \Vert \boldsymbol{\theta }_1 - \boldsymbol{\theta }_2\Vert , \end{aligned}$$

and $\sup _{\boldsymbol{x}_t}\sup _{\boldsymbol{\theta }(\boldsymbol{x}_t)\in \Theta (\boldsymbol{x}_t)} \Vert \boldsymbol{\theta }(\boldsymbol{x}_t)\Vert _{\infty } \le c_b$, where $\Vert \cdot \Vert _{\infty }$ is the vector sup-norm, and $c_b$ and *β * are positive constants.

#### Assumption 2

*(Variance and mean)* For any $\boldsymbol{x}_t$ and some constant $c_v > 0$:

5 $$\begin{aligned} \operatorname {Var}_{\boldsymbol{y}_t|\boldsymbol{x}_t}(\ell (\boldsymbol{\theta }(\boldsymbol{x}_t), \boldsymbol{Y}_t) - \ell (\boldsymbol{\theta }_0(\boldsymbol{x}_t), \boldsymbol{Y}_t)) \le c_v R(\boldsymbol{\theta }_0(\boldsymbol{x}_t), \boldsymbol{\theta }(\boldsymbol{x}_t)), \end{aligned}$$

where $\operatorname {Var}_{\boldsymbol{y}_t|\boldsymbol{x}_t}$ denotes the conditional variance given $\boldsymbol{x}_t$.

#### Theorem 1

(GDP’s excessive risk) Under Assumptions 1–2, the excessive risk is bounded as follows:

6 $$\begin{aligned} \operatorname {E}R(\boldsymbol{\theta }_0, \hat{\boldsymbol{\theta }}) \le \underbrace{c_1\operatorname {E}W(\hat{P}_{\boldsymbol{y}_t|\boldsymbol{x}_t}, P_{\boldsymbol{y}_t|\boldsymbol{x}_t})}_{\text {Generation error}} + \underbrace{c_2m^{-\frac{1}{2}} \log m}_{\text {Synthetic sampling error}}, \end{aligned}$$

where $c_1 = 1+\beta$ and $c_2 = 2^{15} c_v^{1/2}d_{\boldsymbol{\theta }}$, with $d_{\boldsymbol{\theta }}$ denoting the dimension of $\boldsymbol{\theta }$, and $\operatorname {E}$ denotes the expectation with respect to the randomness. Finally, if $\operatorname {E}W(\hat{P}_{\boldsymbol{y}_t|\boldsymbol{x}_t}, P_{\boldsymbol{y}_t|\boldsymbol{x}_t})\le \gamma _n$ with $\gamma _n$ representing the generation error bound in terms of training sample size *n*, then $\operatorname {E}R(\boldsymbol{\theta }_0, \hat{\boldsymbol{\theta }}) \le c_1 \gamma _n + c_2 m^{-\frac{1}{2}} \log m$. As $m \rightarrow \infty$, $\operatorname {E}R(\boldsymbol{\theta }_0, \hat{\boldsymbol{\theta }})\le c_1 \gamma _n$.

Theorem 1 demonstrates that the accuracy of point predictions, derived via risk minimization as defined in (1) on a synthetic sample of size *m*, is determined by two principal components: (i) the generation error inherent in distributional estimation, and (ii) a controllable synthetic sampling error that depends on *m*. The generation error is bounded by the Wasserstein distance, measuring the distributional shift between the synthetic distribution and the data-generating process induced by a specific generator. As *m* increases, the synthetic sampling error decreases, ensuring that the GDP’s prediction accuracy is bounded by the generator’s Wasserstein error.

**Risk minimization.** This result underscores the critical role of precise distribution estimation in risk minimization. A well-estimated distribution inherently facilitates effective risk minimization, enabling optimal performance across a wide range of loss functions. This adaptability marks a paradigm shift: prioritizing accurate distribution estimation enhances downstream predictive performance across various tasks. In Sect. 4.1, we further illustrate the significance of this approach by demonstrating its application to adaptive quantile regression, highlighting its capacity to accommodate diverse predictive objectives.

**Impact of synthetic sample size** *m* **.** Theorem 1 underscores the significance of the synthetic sample size *m* in tightening the bound on GDP’s excess risk. When *m* is sufficiently large, the synthetic sampling error diminishes, leaving the excess risk of GDP upper-bounded by the Wasserstein generation error. Empirical results in Sects. 4.4 and 4.5 support this claim, demonstrating that selecting an appropriate *m* enhances performance compared to the default choice of *m = 1* while effectively managing computational cost. This finding highlights the importance of striking a careful balance between computational efficiency and prediction accuracy, with *m* playing a critical role in this trade-off.

For implementation, this result suggests treating *m* as an inference-time budget. Under a fixed generator, sampler, number of reverse steps, and batching scheme, producing *m* conditional samples for a new input has sampling complexity approximately linear in *m*. Larger *m* reduces finite-sample Monte Carlo variation in the empirical risk but increases sampling time roughly proportionally. A practical choice is to tune *m* on a validation set: start from $m_0$, add samples in increments of *Δ m*, recompute validation loss, and stop when the marginal improvement is below a chosen tolerance or within the validation standard error.

The generation-error term has a different interpretation. It is controlled by how well the fitted generator approximates the conditional response distribution, not by the number of synthetic samples drawn at inference time. Once validation loss is insensitive to further increases in *m*, persistent error is more likely due to generator misspecification, poor calibration, or missing modes than to Monte Carlo variation. Generator adequacy should therefore be assessed on held-out data under the target loss, with task-dependent checks when appropriate, including coverage or quantile calibration for scalar responses, diversity and mode-coverage checks for sampled responses, and semantic consistency checks in a fixed embedding space for text and image outputs. When these diagnostics indicate persistent inadequacy, simply increasing *m* is unlikely to resolve the remaining error; the generator may need calibration, tuning, retraining, or replacement.

## Multimodal Learning and Domain Adaptation

This section focuses on diffusion models, though the GDP framework broadly applies to other generative models.

### Generation via Transfer Learning with Dual-Level Embedding

To enhance target generation, we leverage a source generation task, transferring knowledge from a pre-trained model to improve performance. The transfer process involves aligning the conditional probability distributions of the source $P_(\boldsymbol{y}_s|\boldsymbol{x}_s)$ and the target $P_(\boldsymbol{y}_t|\boldsymbol{x}_t)$ using the dual-level shared embeddings (DSE) framework. This alignment allows effective transfer even when the distributions of $(\boldsymbol{Y}_s, \boldsymbol{X}_s)$ and $(\boldsymbol{Y}_t, \boldsymbol{X}_t)$ differ, facilitating efficient generation with limited target domain data.

The concept of dual-level shared embeddings (DSE) between source and target tasks extends the shared embedding concept for transfer learning in Tian & Shen (2024).

**Dual-level shared embeddings.** Consider an encoder-decoder system, where an encoder * f * maps a random vector $\boldsymbol{Y}_j$ into a latent embedding vector $\boldsymbol{U}_j$, potentially of different dimensionality. A decoder * g * then reconstructs the original data from the embedding $\boldsymbol{U}_j$, ensuring that $\boldsymbol{Y}_j = g(\boldsymbol{U}_j)$ for * j = s, t *. The encoder and decoder act as transformations between the semantic latent space and the observed data space, which is especially important for unstructured data. In an image captioning task, the encoder and decoder translate captions to and from a numerical embedding space. Depending on the styles of captions and photos, the semantic representations of the images may vary in distribution.

The DSE model assumes the conditional distribution of $\boldsymbol{U}_j$ given $\boldsymbol{X}_j$ $P_{\boldsymbol{u}_j|\boldsymbol{x}_j}(\cdot |\boldsymbol{x}_j)$ to follow a shared structure across tasks through an embedding function * h *:

7 $$\begin{aligned} P_{\boldsymbol{u}_j|\boldsymbol{x}_j}(\cdot |\boldsymbol{x}_j) = P_j(\cdot , h(\boldsymbol{x}_j)), \quad j = s, t. \end{aligned}$$

The DSE model in (7) captures the shared structure between source and target tasks. Based on (7), given the pre-estimated $(\hat{f},\hat{g},\hat{h})$ from the source task, we can learn the distribution of $\boldsymbol{U}_t$ in the latent space. Let *ψ * parametrize the conditional generator and $P_t(\boldsymbol{u}_t,h(\boldsymbol{x}_t))$, We estimate the target-specific latent parameter by minimizing the target generation loss using the target samples $\{(\boldsymbol{y}_t^i, \boldsymbol{x}_t^i)\}_{i=1}^{n}$:

8 $$\begin{aligned} \hat{\psi }=\arg \min _{ \psi \in \Psi } \sum _{i=1}^{n} \ell _t \left( \hat{f}\left( \boldsymbol{y}_t^i\right) , \hat{h}(\boldsymbol{x}_t^i); \psi \right) , \end{aligned}$$

where *Ψ * is the parameter space for * ψ *, while $\ell _t$ is the generation loss such as the diffusion score matching loss defined in Appendix C.

Given the learned parameters $(\hat{\psi }, \hat{h})$, the generator produces synthetic samples $(\tilde{\boldsymbol{Y}}_t^i, \boldsymbol{X}_t)_{i=1}^m$ given $\boldsymbol{X}_t$ via the decoder $\hat{g}$. Specifically, it completes the generation process by computing $\tilde{\boldsymbol{Y}}_t^i = \hat{g}(\tilde{\boldsymbol{U}}_t^i)$. Here, we derive $\hat{g}$ from the source task through shared structures and draw the samples $(\tilde{\boldsymbol{U}}_t^i, \boldsymbol{X}_t)_{i=1}^m$ from the distribution $P_t(\cdot ; \hat{h}(\boldsymbol{X}_t), \hat{\psi })$ given $\boldsymbol{X}_t$. The distribution $P_t(\cdot ; \hat{h}(\boldsymbol{X}_t), \hat{\psi })$ denotes the generator’s output distribution parameterized by $\hat{\psi }$.

### Unified Diffusion Generation for Tabular and Unstructured Data

This subsection describes a conditional diffusion implementation for synthesizing multimodal samples by combining structured tabular covariates with unstructured information, such as text or image embeddings. Diffusion models have shown strong performance for unstructured data (Ho et al., 2020; Song et al., 2021) and tabular data (Kotelnikov et al., 2023); here, we use shared covariate embeddings and a shared response encoder–decoder (Sect. 3.1) as one task-adapted way to connect these data types within the GDP framework. The implementation supports transfer learning between source and target domains while allowing modality-specific embedding layers and tunable embedding sizes for downstream tasks.

**Fig. 1:**
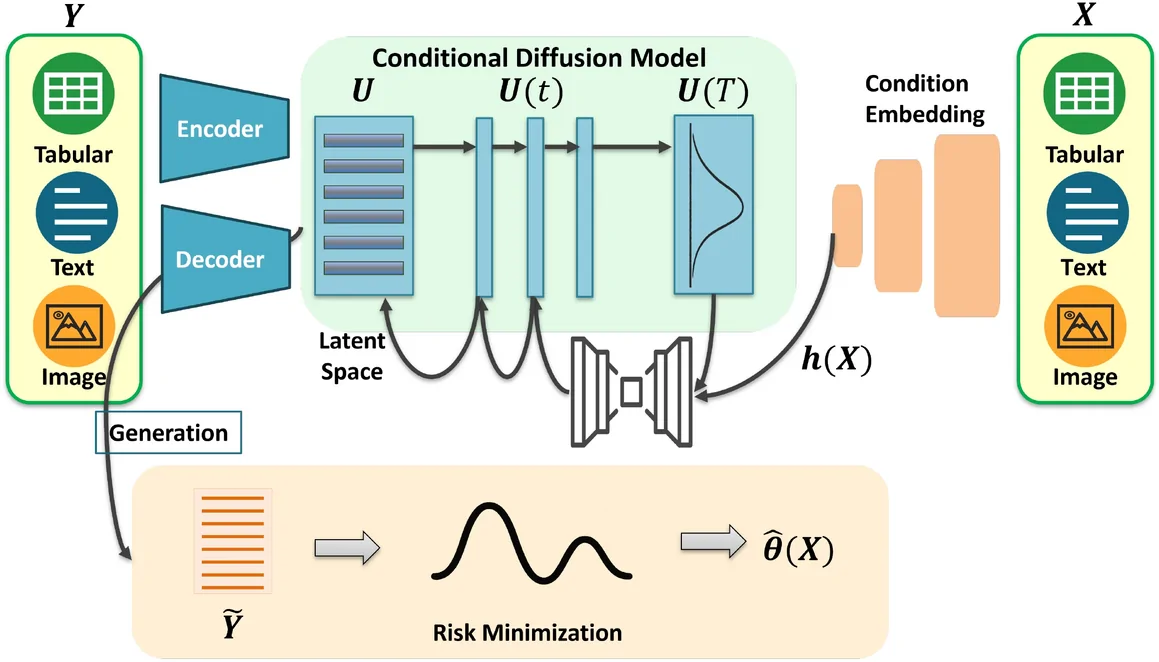
Conditional diffusion models for target domain adaptation through shared representations

Figure 1 illustrates this implementation, which uses both structured (tabular) and unstructured (text or image) data for transfer learning. Dedicated encoders convert each modality into numerical embeddings; separate condition embeddings are then combined into a shared representation used by the conditional diffusion model. The conditioning mechanism provides input-specific context during generation, while the encoder–decoder maps response variables into a latent space and reconstructs generated representations into their original formats when needed. Key components of the model include shared neural networks (*f*, *g*, *h*) trained on source data. This sharing encourages feature transfer across domains while still allowing task-specific adaptation.

Algorithm 1 outlines the complete GDP procedure step by step. In summary, this diffusion model efficiently supports transfer learning by utilizing shared representations and multimodal embeddings, making it ideal for tasks that require integrating structured and unstructured data across different domains.

**Algorithm 1:**
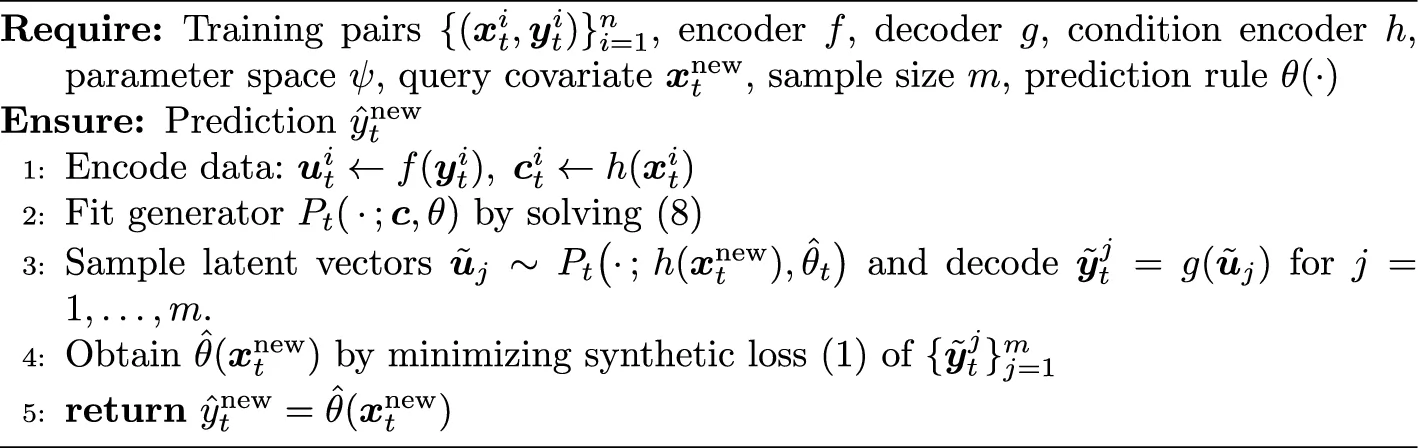
Generative Distribution Prediction (GDP) via Transfer Learning

## Numerical Examples

This section evaluates GDP in tabular, image, and text prediction tasks by assessing predictive accuracy and comparing it with standard and task-specific baselines on benchmark datasets and simulated examples. We examine several variants of GDP, including diffusion-GDP (which incorporates diffusion generation with a statistical guarantee), GDP integrated with large pre-trained models, and transfer-GDP enhanced through transfer learning.

Our evaluation encompasses four supervised tasks that yield tabular and text outputs using a combination of tabular, text, and image predictors. Specifically, Subsects. 4.1–4.5 detail experiments: (1) adaptive quantile regression across multiple quantiles using diffusion models; (2) modal regression via diffusion models; (3) Tabular prediction with multimodal predictors for regression and classification tasks; (4) image captioning with diffusion models; (5) question answering utilizing a large language model.

A detailed description of numerical experiments is provided in Appendix B. The code used to generate the numerical results is publicly available at https://github.com/shakayoyo/GDP.

### Diffusion-GDP for Adaptive Quantile Regression

**Quantile regression** estimates the conditional *α *-th quantile of the response for a specified quantile level $\alpha \in (0,1)$, by minimizing the asymmetric pinball loss (Koenker & Bassett, 1978; Steinwart & Christmann, 2011). Recent deep learning approaches have achieved state-of-the-art performance in semiparametric and nonparametric quantile estimation (Zhong et al., 2022, 2021; Zhong & Wang, 2024), in contrast to classical methods that typically impose restrictive model assumptions for estimation and prediction.

In this section, we examine how a generative approach such as GDP, which directly models the conditional distribution of the response given the predictors, can improve estimation across a range of quantile levels *α *. Compared with supervised methods trained for a single, fixed quantile, the generative framework can reuse the same fitted conditional distribution for different quantile targets, providing a flexible approach to conditional quantile estimation.

This experiment considers nonparametric quantile regression via simulations to evaluate the performance of diffusion-GDP for a prediction task, where the true data-generating mechanism is known. Specifically, let $\boldsymbol{X}= (X_1, \ldots , X_p)^T$ be a vector of predictors, and define the response *Y* as

9 $$\begin{aligned} Y = \sin (\textbf{X}^T \boldsymbol{\beta }) \;+\; \log \bigl (1 + |X_1|\bigr ) \;+\; \epsilon \,(1 + \,|X_2|), \end{aligned}$$

where $\boldsymbol{X}^T$ denotes the transpose of $\boldsymbol{X}$, and $\boldsymbol{\beta }$ is the vector of regression coefficients generated uniformly in *(-1,1)*. To induce heteroscedasticity, the noise term *ε * follows a normal distribution with mean 0 and variance $|X_2|$, that is, $\epsilon \sim N\bigl (0, |X_2|\bigr )$. Hence, the variance is modulated by the magnitude of $|X_2|$. This setup naturally creates a challenging scenario for regression, incorporating both nonlinear and heteroscedastic behavior. Two marginal distributions for $\boldsymbol{X}$ are considered:

- **Case I:** $\boldsymbol{X}\sim N(\boldsymbol{0}, \boldsymbol{I})$, where $\boldsymbol{I}$ is the identity matrix.
- **Case II:** $X \sim N(\boldsymbol{0}, \boldsymbol{\Sigma })$, where the covariance matrix $\boldsymbol{\Sigma }$ is defined by $\Sigma _{ij} = \rho ^{|i-j|}$ with * ρ = -0.5 *. Here $\boldsymbol{\Sigma }=(\Sigma _{ij})_{p \times p}$ follows an AR(1) dependence structure and 1 is the common variance of each $X_i$ and *ρ * (*|ρ | <1*) is the lag-1 correlation between two observations $X_i$ and $X_{i+1}$.

In this study, we focus on quantile adaptation by evaluating the predictive performance of several supervised models across the 5%, 20%, 50%, 80%, and 95% quantiles. While a single supervised model may perform well at predicting a specific quantile, it often fails to deliver consistent accuracy across a broader range of quantiles.

For diffusion-GDP, we employ a Gaussian diffusion model that offers statistical guarantees (see Theorem 1 and Corollary 1 in Appendix C). We compare its performance against two state-of-the-art methods—extreme gradient boosting (XGBoost; Chen & Guestrin, (2016)) and deep quantile regression (DQR; Padilla et al., (2022)). Each model is designed to predict multiple quantiles of $\boldsymbol{Y}$ given $\boldsymbol{x}$.

In our simulations, we generate $n = 10{,}000$ observations with *p = 100* based on the model in (9) for Cases I and II. For quantile regression, GDP trains the diffusion model to perform conditional generation of $\boldsymbol{Y}$ given $\boldsymbol{x}$ using the conditional likelihood with $m = 1{,}000$ samples, following the procedure described in Section 2.1. Additionally, XGBoost and DQR optimize using the averaged pinball loss over the target quantiles of 5%, 20%, 50%, 80%, and 95%.

The simulated data are split into training and test datasets with a ratio of 7:3. For the testing data, we compute the quantiles as the truth for each $\boldsymbol{x}$ by sampling *y* from the true model. For evaluation, we use Root Mean Squared Error (RMSE) between the estimated quantiles and the true quantiles and Pinball loss based on the observed responses, computed at each quantile level, and report the overall performance averaged across the five quantile levels.

As shown in Table 1, GDP consistently achieves lower RMSE than DQR and XGBoost, whether the evaluation metric is computed over the averaged quantile loss (over 5%, 20%, 50%, 80%, and 95%) or at the tail 95% quantile. Although the magnitude of GDP’s advantage varies across cases—sometimes only marginal—every difference is statistically significant at the $\alpha =5\%$ level. This accuracy boost comes at the cost of 4-5 times longer but manageable computation times for our experimental scale.

These findings highlight the benefit of modeling the entire conditional distribution. By capturing a richer distributional structure, GDP aligns more closely with risk-oriented metrics such as RMSE. While XGBoost’s gradient-boosted trees excel on structured (and even sparse) data and DQR directly models nonlinearities and heteroscedasticity through quantile regression, GDP gains a further edge by training on a distributional loss rather than the pinball loss, enabling it to capture broader distributional nuances and deliver superior predictive performance.

**Fig. 2:**
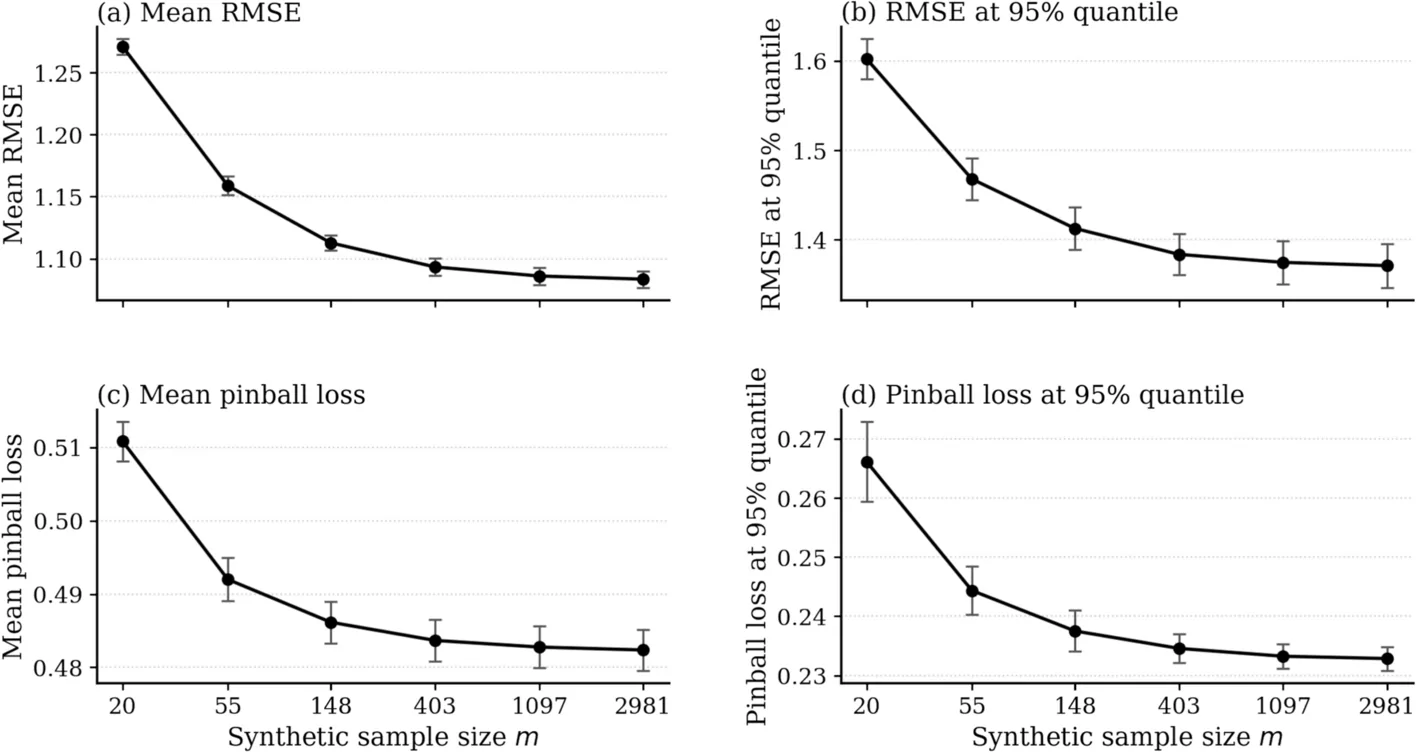
Sensitivity of GDP to the number of synthetic samples *m* in the adaptive quantile regression experiment. Points report averages over five random replicates, and error bars denote one standard error. The tested values of *m* are approximately exponentially spaced

To assess the sensitivity of GDP to the number of synthetic samples used at inference, we further conduct an ablation study for Case I. We fix the trained GDP architecture with time-embedding dimension 64, condition-embedding dimension 32, hidden dimension 256, and 5 residual blocks, and vary only the inference-time synthetic sample size *m*. Specifically, we use $m=\textrm{round}\{\exp (k)\}$ for *k=3,… ,8* and average the results over five random replicates. Figure 2 reports the mean RMSE, RMSE at the 95% quantile, mean pinball loss, and pinball loss at the 95% quantile. The errors decrease as *m* increases and then stabilize, indicating diminishing returns from additional synthetic samples. This pattern is consistent with Theorem 1: increasing *m* reduces the synthetic sampling error, whereas the remaining performance is governed by the generation error of the fitted conditional diffusion model. The value *m=1000* used in the main comparison is therefore within the stable region of the accuracy–efficiency trade-off. This empirical stabilization supports the validation-based marginal-gain rule for selecting *m* discussed after Theorem 1.

**Table 1:** RMSE and pinball loss (overall and at the 95% quantile)

Metric	Case I			Case II		
	GDP	DQR	XGBoost	GDP	DQR	XGBoost
RMSE 95%	1.678	1.826 (0.000)	2.083 (0.000)	1.847	2.084 (0.028)	2.195 (0.002)
RMSE Avg.	1.139	1.193 (0.009)	1.345 (0.000)	1.191	1.279 (0.022)	1.342 (0.003)
Pinball 95%	0.223	0.225 (0.007)	0.237 (0.000)	0.226	0.235 (0.013)	0.247 (0.000)
Pinball Avg.	0.472	0.473 (0.002)	0.483 (0.000)	0.473	0.477 (0.014)	0.483 (0.000)
Time (s)	903.07	182.29	184.97	840.20	194.45	183.18

Runtimes are listed in the bottom row. Paired *t*-test *p*-values (in parentheses) show GDP’s advantage over DQR and XGBoost

### Diffusion-GDP for Modal Regression

**Modal regression** aims to estimate the mode of the conditional distribution of the response variable *Y* given the predictors $\boldsymbol{X}$. Assuming a one-dimensional response *Y* for simplicity, the true modal regression function, $\theta _0(\boldsymbol{x})$, is defined as the value that maximizes the conditional probability density function (PDF): $\theta _0(\textbf{x})={\mathrm{arg\, max}}_{\theta }p_{y|\boldsymbol{x}}(\theta |\boldsymbol{x}).$

In this experiment, we evaluate the diffusion-based GDP method against a kernel-based modal regression baseline (Chen et al., 2016) for mode estimation across two nonlinear data-generating mechanisms. In both scenarios, the covariates are two-dimensional and drawn from a uniform distribution: $\boldsymbol{X}= (X_{1}, X_{2})^\top$, with $X_{1}, X_{2} \sim \textrm{Unif}(-2, 2)$ independently. The response is generated as $Y = f(\boldsymbol{X}) + a(\boldsymbol{X})\,\epsilon$, with $\epsilon \sim \textrm{Exp}(1) - 1$, where *f(· )* is a nonlinear signal function and *a(· )* is a scale function controlling the noise level.

**Case I: Homoskedastic exponential noise.** In the first case, we set a constant noise scale $a(\boldsymbol{x}) \equiv 1$ and choose $f(\boldsymbol{X}) = 2\sin (\pi X_{1}) + X_{2}^2$, so that $Y = f(\boldsymbol{X}) + \epsilon .$ Since *ε * has mean zero and mode *-1*, the true conditional mode in this case is $\theta _0(\boldsymbol{x}) = 2\sin (\pi x_1) + x_2^2 - 1$, which is nonlinear in both coordinates of $\boldsymbol{x}$ but has a homoskedastic conditional distribution of *Y* given $\boldsymbol{X}$.

**Case II: Heteroskedastic noise.** In the second, more challenging case, we allow the noise scale to depend on the covariates. Specifically, we define $f(\boldsymbol{X}) = 1.5 \sin (\pi X_{1}) + 0.5 X_{1}^2 + X_{2}^2$, and $a(\boldsymbol{X}) = 0.5 + 0.5 |X_{1}| + 0.3 X_{2}^2$. Again using that $\mathbb {E}(\epsilon )=0$ and $\textrm{mode}(\epsilon )=-1$, we obtain that the true conditional mode in this case is

$$ \theta _0(\boldsymbol{x}) = 1.5 \sin (\pi x_1) + 0.5 x_1^2 + x_2^2 - \bigl (0.5 + 0.5 |x_1| + 0.3 x_2^2 \bigr ), $$

which remains nonlinear in both coordinates but now exhibits covariate-dependent spread and skewness in the conditional distribution of $Y \mid \boldsymbol{X}=\boldsymbol{x}$. This heteroskedastic case is designed to test the flexibility of both the diffusion-based and kernel-based modal regression methods. Each repetition uses $n_{\text {train}} = 1{,}500$ training pairs and $n_{\text {test}} = 500$ test pairs generated independently from this data-generating mechanism.

In the GDP method, we generate $m = 1{,}000$ conditional samples for each test point from the trained diffusion model. To estimate the conditional mode, we apply a univariate kernel density estimator (KDE) independently for each covariate value $\textbf{x}$. Specifically, for a test point $\textbf{x}$ with generated samples $\{\tilde{Y}^{k}\}_{k=1}^m$, we construct a Gaussian KDE on a fine evaluation grid, using a bandwidth selected by standard rule-of-thumb methods. The GDP mode estimator is then defined as $\hat{\theta }(\textbf{x}) = \arg \min _{\theta }\sum _{k=1}^m \ell (\theta ,\tilde{Y}^{k})$ with $\ell (\theta ,\tilde{Y}^{k}) = \mathcal {K}_{\sigma }(\theta - \tilde{Y}^{k})$, where $\mathcal {K}_{\sigma }$ denotes the Gaussian kernel with bandwidth *σ *.

As summarized in Table 2, the conditional-diffusion-based GDP achieves lower estimation errors than the classical kernel-based modal regression method (RegMS2d, https://github.com/yenchic/ModalRegression) in both simulation settings. Specifically, GDP delivers a 57% improvement in Case I and a 37% improvement in Case II relative to RegMS2d. These substantial gains highlight the accuracy and robustness of GDP in estimating conditional modes, particularly under complex nonlinear or heteroskedastic data-generating processes.

**Table 2:** Means (standard deviations in parentheses) of RMSEs for conditional mode estimation over 100 repetitions in the two simulation cases

Method	Case I	Case II
GDP	0.5039 (0.0598)	0.6498 (0.0751)
RegMS2d	1.1912 (0.1328)	1.0311 (0.1208)

### Diffusion-GDP for Tabular Prediction with Multimodal Predictors

This subsection benchmarks our GDP approach—which integrates tabular and unstructured inputs—against strong baselines on both classification and regression tasks.

- Yelp Review (Multi-class). From the public Yelp Dataset,^1^ a benchmark for star-rating prediction, we sample 100,000 reviews (1–5 stars). To create domain shift, reviews with zero "cool" votes form the source split (79,984) and those with at least one vote form the target split (20,016). The random split is 80/20 into train/test; we predict star ratings from review text and metadata. To respect the ordinal nature of the ratings, we assess Yelp review predictions using Cohen’s *κ * (Cohen, 1960), a chance-corrected agreement statistic that is robust to class imbalance and tailored to ordinal categories such as star ratings.
- UTKFace (Regression). UTKFace dataset^2^ contains 23,705 face images labeled with age, gender, and ethnicity. We apply an 80/20 random train/test split and predict the age of each image. UTKFace prediction is evaluated with root mean square error (RMSE).
- Shopee-IET (Multi-class). The Shopee-IET (Shopee Image-based E-commerce Text retrieval) dataset comprises images of clothing items spanning 18 categories. In this experiment, we utilize the four-category subset from AutoGluon’s benchmark collection,^3^ which contains 1000 images. We apply an 80/20 random train/test split and train a model to predict each image’s category label. Model performance is evaluated using classification accuracy.

*Tabular baselines with feature generation*: CatBoost (CB, Prokhorenkova et al. (2018)), XGBoost (XGB, Chen & Guestrin, (2016)), and a fully connected neural network (NN). All three are supervised models for tabular prediction. For Yelp, we convert review text to tabular features with AutoGluon’s tabular feature generator Erickson et al. (2020) (AutoGluon is an open-source AutoML toolkit).

*Multimodal baseline*: Multimodal Predictor (AutoMM) from AutoGluon library (Tang et al., 2024), a supervised model trained end-to-end to predict the target labels, configured with an Electra encoder for text (Clark et al., 2020) and a ResNet encoder for images (He et al., 2016).

*Transformer baselines*: we include the Tabular-Text Transformer (TTT; Bonnier, 2024) for the Yelp text-tabular task and TIP (Du et al., 2024) for the UTKFace image-tabular task. These baselines provide recent transformer-based multimodal predictors tailored to the corresponding data types.

*Distribution-aware probabilistic baselines*: we include NGBoost (Duan et al., 2020) and a Bayesian neural network (BNN) baseline implemented with Bayesian-Torch (Krishnan et al., 2022). For Yelp and UTKFace, these methods are trained on fixed text–tabular and image–tabular features, respectively; for Shopee-IET, they are trained on the same fixed AutoMM image embeddings and evaluated on the same five data splits. These baselines provide direct comparisons with probabilistic prediction methods that estimate predictive distributions rather than only point labels. Their reported runtimes measure only the downstream probabilistic model fitting on the fixed features or embeddings, excluding upstream feature-extractor training and embedding extraction.

We also evaluate a domain-adapted variant of GDP (GDP-tr) on Yelp, in which we pretrain the condition encoder on the source set (79,984 non-enthusiastic reviews) before fine-tuning the target training split. For fairness, the AutoMM and GDP-based multimodal models use the same Electra text backbone and ResNet image backbone when applicable, while TTT and TIP retain their own transformer-style fusion architectures.

Each experiment is repeated 5 times and the average results are reported in Table 3.

**Table 3:** Average performance and runtime across five random train/test splits

Data type	Pred	Metric	CB	XGB	NN	AutoMM	NGBoost	BNN	TTT	TIP	GDP	GDP-tr
Yelp review multiclass	Text	Cohen’s *κ *	0.361 (0.000)	0.3541 (0.000)	0.111 (0.000)	0.495 (0.170)	0.303 (0.000)	0.421 (0.000)	0.418 (0.000)	NA	0.483	**0**.**520** (0.000)
		Time (s)	202.50	88.48	37.46	922.92	5502.41	28.08	317.92	NA	528.45	629.26
UTKFace regression	Image Tabular	RMSE	19.077 (0.000)	19.077 (0.000)	19.219 (0.000)	10.549 (0.000)	11.962 (0.000)	22.822 (0.000)	NA	7.988 (0.017)	**7**.**511**	NA
		Time (s)	10.90	84.27	64.25	1776.12	792.77	12.94	NA	614.09	5705.93	NA
Shopee-IET multiclass	Image	Accuracy	NA	NA	NA	0.872 (0.076)	0.876 (0.078)	0.878 (0.078)	NA	NA	**0**.**944**	NA
		Time (s)	NA	NA	NA	145.42	10.46	0.65	NA	NA	178.68	NA

Numbers in parentheses are *p*-values from paired *t*-tests of GDP’s performance against each method. Wall-clock training times (in seconds) are shown in the bottom row. “NA” indicates that a method does not apply to the dataset. Bold values indicate the best predictive performance for each dataset among applicable methods

Across the three benchmarks, the base GDP model attains the highest accuracy on UTKFace and Shopee-IET—the two datasets where GDP-tr is not applicable. On Yelp, GDP falls just short of AutoMM, yet the transfer-enhanced variant (GDP-tr) overtakes every competitor and secures the top overall score. The transformer baselines are also competitive: TTT improves substantially over the tabular-only Yelp baselines but remains below AutoMM, GDP, and GDP-tr, while TIP substantially improves over AutoMM on UTKFace and comes close to GDP. The probabilistic baselines provide useful distribution-aware comparisons, but they do not close the gap to GDP on the image benchmarks.

**Yelp text-rating.** GDP improves Cohen’s *κ * by 34% over CatBoost, 36% over XGBoost, and more than 400% over a plain neural network. TTT reaches 0.418, outperforming the tabular-only methods but remaining below AutoMM (0.495), GDP (0.483), and GDP-tr (0.520); the paired test against GDP is significant (*p < 0.001*). The BNN baseline reaches a similar level (0.421), whereas NGBoost is lower (0.303). Transferring from GDP to GDP-tr provides an additional 5% improvement over the strong multimodal baseline AutoMM (*p < 0.001*), even though GDP alone performs comparably to AutoMM (*p > 0.05*).

**UTKFace age regression.** GDP reduces the RMSE from 10.55 (AutoMM) to 7.51, a 29% improvement (*p < 0.001*). TIP attains an RMSE of 7.99, substantially improving over AutoMM and far outperforming the tabular-only methods, while remaining slightly above GDP; the paired test against GDP gives *p = 0.017*. NGBoost improves over the tabular-only baselines but remains behind AutoMM, TIP, and GDP (RMSE 11.96), while the BNN baseline has higher error in this setting (RMSE 22.82). Other tabular methods perform poorly in this experiment, as only gender and ethnicity are available as predictors.

**Shopee-IET image classification.** GDP improves AutoMM’s classification accuracy from 0.872 to 0.944, an absolute increase of 7.2 percentage points (8% relative). NGBoost and BNN, trained on the same fixed AutoMM image embeddings, obtain accuracies of 0.876 and 0.878, respectively, placing them close to AutoMM but still below GDP. Although GDP’s improvement over AutoMM narrowly misses statistical significance at the 5% level (*p = 0.076*), it corresponds to roughly 10 additional correct predictions per 125 images.

All experiments were run on the same compute node equipped with an NVIDIA Tesla V100 PCIe GPU (32 GB). The average runtime for each method is reported in Table 3. Regarding computational cost, the GDP method may generally incur longer runtimes due to its sampling procedure of the diffusion model—especially when the synthetic sample size *m* is large—illustrating the accuracy–efficiency trade-off. In practice, however, its overhead remains comparable to that of the multimodal method of AutoMM. Overall, GDP’s accuracy gains come at a reasonable computational cost.

### Applying GDP with Diffusion and Large Pre-trained Models to Image Captioning

This subsection evaluates the performance of the GDP method within a multimodal framework for image captioning. Specifically, we integrate GDP with two distinct multimodal generators—a diffusion model and the Bootstrapping Language-Image Pre-training (BLIP) model (Li et al., 2022)—yielding the diffusion-GDP and BLIP-GDP variants, respectively. Notably, the diffusion-GDP approach is supported by the theoretical results presented in Theorems 1 and 2 in the Appendix, whereas no corresponding theory currently exists for BLIP-GDP.

Modern image captioning typically employs an encoder-decoder framework. A Convolutional Neural Network (CNN) extracts image features then passes to a decoder—originally an LSTM model (Vinyals et al., 2015) and more recently a Transformer—to generate captions such as BLIP involving attention mechanisms.

For our experiments, we employ the captions_val2014 subset of the widely recognized COCO Caption dataset (available at http://coco-dataset.org). We use the first 35,000 samples for training and reserve the remaining 5,504 samples for testing. The COCO Caption dataset is a standard benchmark for image captioning, featuring richly annotated images paired with human-generated captions. Specifically, we train our diffusion model on the designated training set, while we do not fine-tune BLIP since it was pre-trained on the entire COCO Caption dataset. We evaluate both methods on the 5,504 test samples.

In our multimodal diffusion model, as described in Sect. 3.2, we use SentenceBERT (Sentence-Bidirectional Encoder Representations from Transformers) (Reimers, 2019) to extract sentence embeddings and CLIP (Contrastive Language-Image Pretraining) (Radford et al., 2021) to align textual and visual modalities, together forming the encoder. We then apply a Gaussian diffusion model in the embedding space, leveraging a residual network (He et al., 2016) to enhance feature representation and maintain model stability. Finally, we use a pre-trained Vec2Text model (Morris et al., 2024) to decode the diffused vector embeddings to generate image captions.

To assess alignments between the generated and reference captions, we compute cosine similarity scores between their sentence embeddings, where we obtain these embeddings using models such as Google’s text-embedding-004 (Google, 2025) via APIs. The score ranges from 0 to 1, with 1 indicating complete semantic equivalence. For each image in the test set, each generator produces *m=10* candidate captions. The GDP method selects the final caption by minimizing the cosine dissimilarity loss among 10 candidates, defined as one minus the cosine similarity score, over these candidates, as specified in (1). Specifically, we adopt the expected semantic dissimilarity minimizer as our prediction rule for selecting the most appropriate caption. Formally, this is defined as

$$ \theta = {\mathop {{\mathrm{arg\, min}}}\limits _{\boldsymbol{y}'}}\, \mathbb {E}_{\boldsymbol{y}|\boldsymbol{x}}\bigl [d(\boldsymbol{y},\boldsymbol{y}')\bigr ], $$

where *d* denotes the cosine dissimilarity. In practice, we approximate $\mathbb {E}_{\boldsymbol{y}|\boldsymbol{x}}[d(\boldsymbol{y},\boldsymbol{y}')]$ using the empirical average of cosine dissimilarities between each candidate caption $\boldsymbol{y}'$ and the sampled captions $\boldsymbol{y}$ conditioned on a given image $\boldsymbol{x}$. To address verifier-style reranking baselines, we also evaluate a CLIP-based reranker using the same generated candidate pools. For each image, the reranker selects the candidate caption with the largest pretrained CLIP image–text alignment score (Radford et al., 2021); reference captions are used only for final evaluation, not for selection. Finally, we compare the captions selected by our method and by CLIP reranking to the average performance of all candidate captions generated by BLIP and the diffusion model, using their cosine similarity scores with the reference captions as an evaluation metric.

**Fig. 3:**
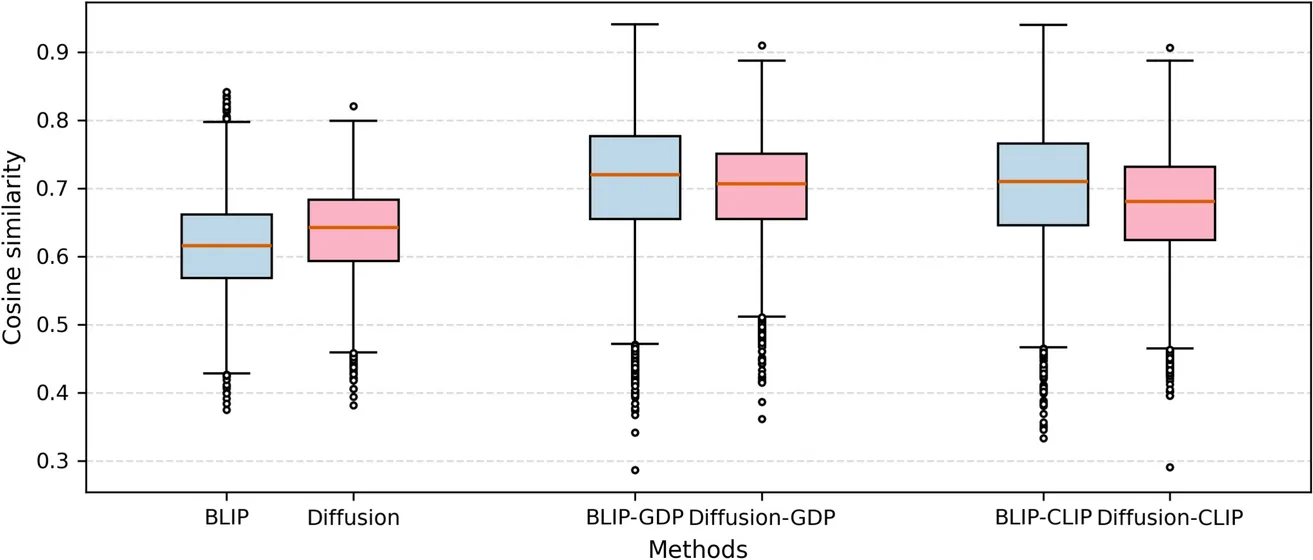
Cosine similarity scores on the COCO caption test set of 5504 images. BLIP and Diffusion report the average similarity over *m=10* generated candidate captions per image. BLIP-GDP and Diffusion-GDP select captions by minimizing empirical semantic dissimilarity within the same candidate pool. BLIP-CLIP and Diffusion-CLIP select captions using a CLIP-based image–text alignment reranker. All six methods are evaluated by reference-caption embedding similarity

Figure 3 shows that our multimodal diffusion model (mean: 0.6372, median: 0.6420) performs comparably to the pre-trained BLIP model (mean: 0.6148, median: 0.6153) when averaging 10 generated captions per image—even though BLIP uses the entire COCO data and additional external data while our diffusion model relies solely on training data. Moreover, after applying GDP selection, the diffusion-GDP and BLIP-GDP methods significantly improve (mean scores of 0.6994 and 0.7099 and median scores of 0.7062 and 0.7203, respectively). The CLIP-rerank baseline improves upon the raw candidate average for both generators, achieving mean scores of 0.6746 for diffusion-CLIP and 0.7006 for BLIP-CLIP; however, GDP selection remains the stronger method on both candidate sets, with paired tests indicating *p<0.001* in both comparisons. This result demonstrates that the GDP selection, based on risk minimization, consistently chooses captions that better align with the reference, providing a clear advantage over random selection and a competitive advantage over an external learned image–text verifier.

In Table 4, an example illustrates how GDP enhances captioning. The GDP method selects captions with high similarity scores generated by diffusion, ensuring better alignment with the image content, while lower scores help filter out low-quality captions that fail to accurately depict the pasta dish in the example image.

**Table 4:** Example of the GDP method in image captioning

Image	Diffusion-generated captions	Similarity (Generation)	Similarity (Reference)
	On a plate you will see a serving of pasta with vegetables on the top	**0.6773**	**0.6675**
	a pasta dish with broccoli, bruzzelli, and serafo sauce on the table. You can see the texture and the slime	0.6317	0.6094
	A dish of pizza on a plate containing a bowl of vegetables (Bisseta, mash, and cheese) topped.	0.6291	0.5743
	A pasta containing a salad with some vegetables (chow and fruit) and some vegetables (snappe and mashi) is sticking	0.6333	0.5968
Reference captions	An eating platter of grilled cheese is inside a nasty and full of sliced grass and grilled potato.	0.5811	0.5230
A large bowl full of noodles and onions	Pasta with a bowl of noodles on a table. Smoothie pasta and sauce from the pasta bowl have been enjoyed for several months	0.6267	0.6452
A bowl filled with pasta and vegetables sitting on a gray counter.	Vegetables serving salad dressed with broccoli, cheese, sardines, pepper, and fruit. trimmed down	0.6393	0.6181
A bowl of pasta salad with onions and olives.	An assemblage of a bowl of noodles. A few bowls of pepper, chili, and sliced vegetables can be enjoyed for a few minutes.	0.6344	0.6617
A corkscrew pasta salad with onions and broccoli.	A plate of pasta in a pan topped with potatoes and mushrooms. A plate of pasta topped with chilies and parboiled potatoes.	0.6463	0.6186
A close-up of a bowl of food with broccoli noodle salad and onions	a sloppy pasta with vegetables on the top and a salad of broccoli and nausea (cook-nose).	0.6223	0.5916

“Similarity (Generation)” represents the average similarity score between a generated caption and nine others from the diffusion model, and "Similarity (Reference)" denotes the average similarity score between a generated caption and five reference captions. The best-selected caption from 10 candidates is bolded

### Applying Large Language Model-GDP to Q&A

This subsection examines the GDP’s performance in question-answering (Q&A) tasks, where both questions and their corresponding answers are textual. The analysis evaluates the accuracy of responses generated by four methods using the LLaMA−3.1-8B-Instruct model (Aimeta, 2024) within a Q&A framework, leveraging the widely recognized WikiQA dataset (Yang et al., 2015). The WikiQA corpus is a benchmark dataset for open-domain Q&A tasks and consists of 3,047 questions sourced from user queries submitted to Bing, paired with 29,258 candidate answers extracted from Wikipedia sentences. Among these, 1,473 question-answer pairs are labeled as correct, indicating an alignment between the questions and their respective answers.

In this experiment, we randomly sample 100 question-answer pairs from the subset of pairs labeled as correct to serve as the test set. This sampling ensures that the evaluation focuses on instances with human-validated answers based on dataset annotations.

To evaluate the semantic similarity between two text answers, we compute the cosine similarity score between their sentence embeddings, following the same approach described in Sect. 4.4.

We evaluate four methods using the LLaMA−3.1-8B-Instruct model (Aimeta, 2024) to answer questions, with the temperature parameter controlling the randomness or diversity of the model’s outputs. The default temperature is set to 0.7, balancing diversity and determinism. The first method is the GDP approach, which employs a cosine dissimilarity loss (1 minus the cosine similarity score) applied to answers generated by the LLaMA model, as defined in (1) with *m=50*, where the prediction rule is as in the previous example. This generative approach selects the answer that minimizes the dissimilarity loss averaged over *m=50* generated answers for the same question, prioritizing semantic similarity to the correct answer. The second method is the deterministic approach, which uses the LLaMA model with a temperature setting of 0. This configuration ensures that the model always returns the same answer for a given question, emphasizing consistency and accuracy in output. The third method is another generative approach using the default temperature setting of 0.7 without minimizing a loss function over *m=50* candidates. Unlike the GDP approach, it averages performance over the *m=50* generated answers for each question, introducing diversity in the outputs. The fourth method is an LLM-as-a-judge baseline (Zheng et al., 2023), where a Gemini Flash judge model selects one answer from the same *m=50* candidate set using the question and candidate answers only; the reference answer is used solely for final evaluation.

**Fig. 4:**
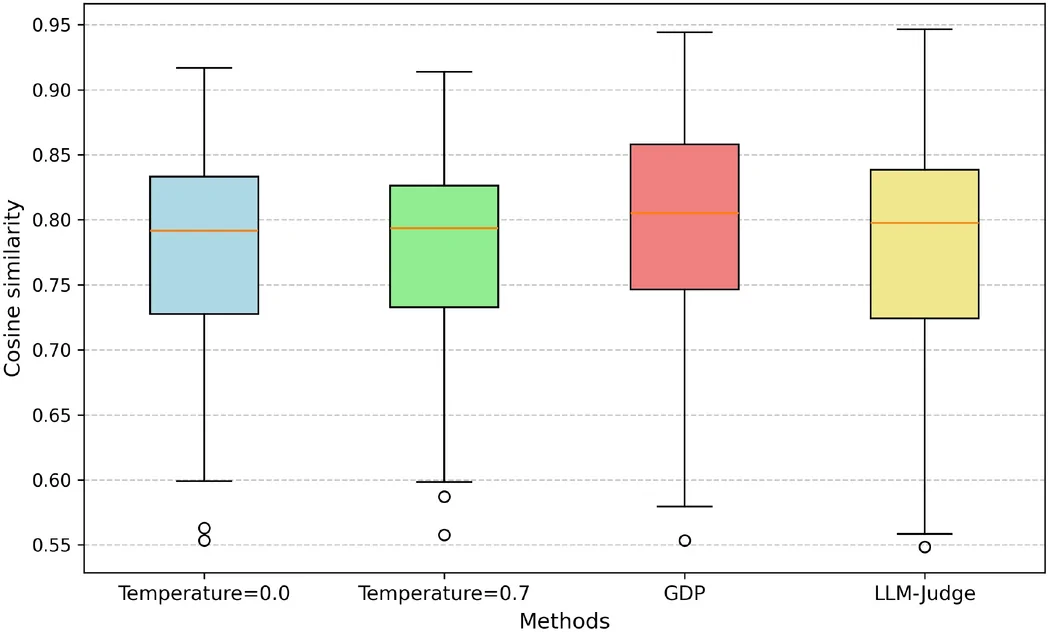
Comparative boxplots of cosine similarity scores for four question-answering methods over the test sample of size 100. ”Temperature=0.0,” ”Temperature=0.7,” and ”GDP” represent the deterministic, generative, and GDP approaches, respectively, based on answers generated by the LLaMA−3.1-8B-Instruct model for a given question. "LLM-Judge" selects one answer from the same candidate set using a Gemini Flash verifier model

As shown in Fig. 4, the mean similarity scores for the deterministic, generative, GDP, and LLM-Judge methods on the test set are 0.7796, 0.7762, 0.7935, and 0.7789, respectively, while the corresponding median values are 0.7915, 0.7934, 0.8052, and 0.7974. These results indicate that the GDP method enhances the performance of the generative model and remains competitive with a modern verifier-style LLM-judge selection baseline.

This systematic comparison underscores the impact of the synthetic sample size *m* and the inherent trade-offs between accuracy, diversity, and selection criteria. When generating a single answer, the deterministic approach often surpasses the generative approach (using the default temperature) by prioritizing accuracy. In contrast, GDP effectively manages this trade-off by optimizing the final output from a diverse yet comparable set of candidate answers. The LLM-Judge baseline also selects from the same candidate set, but it relies on an external learned verifier rather than the semantic-consensus risk minimization used by GDP. This approach balances accuracy and diversity, aligning with the discussion on the impacts of synthetic sample size for GDP in Theorem 1.

## Discussion

The proposed Generative Distribution Prediction framework provides a conceptually unified distributional approach to multimodal learning: it learns or approximates a conditional response distribution and then derives task-specific predictions through loss-adapted risk minimization. Across applications, the encoders, losses, and generative backbones may be chosen to match the modality and task. Transfer learning and conditional generation can improve the fitted conditional distribution when the generator is well matched to the target problem, and the resulting synthetic samples can support uncertainty quantification and loss-adapted prediction. The strength of these benefits depends on generator fidelity and should be assessed through validation for the target task.

Experimental results across various tasks, including tabular prediction, image captioning, question answering, quantile regression, and modal regression, show that GDP and its transfer-enhanced variant achieve competitive or improved performance relative to traditional and strong task-specific baselines in the studied settings. These results suggest that modeling the conditional distribution and applying loss-adapted decision rules can reduce prediction errors and improve robustness, while the degree of improvement depends on the modality, baseline, and generative model quality.

The theoretical foundation developed in this paper provides statistical guarantees for diffusion-based GDP. Recent work (Tian & Shen, 2024) suggests that related guarantees may also be possible for normalizing flow models. However, establishing comparable generation-accuracy theory for large and highly complex generative models, such as BLIP and modern LLMs, remains an open problem. Thus, the image-captioning and Q&A experiments should be interpreted as empirical demonstrations of GDP’s practical flexibility, whereas the formal statistical guarantees in this paper are established for diffusion-based GDP. This limitation motivates further theoretical work on GDP with different classes of generative models.

For practical use, two issues are especially important. First, GDP should be used with a generator that is adequate for the target decision problem. As discussed after Theorem 1, held-out validation under the target loss can help separate finite-sampling error from generator misspecification: if validation performance has stabilized with respect to *m* but task-specific checks still indicate poor calibration, limited coverage, hallucination, or missing modes, simply increasing *m* is unlikely to resolve the remaining error, so generator calibration, tuning, retraining, or replacement may be needed. Second, GDP has an inference-time cost because it draws conditional samples. Since sampling cost is approximately linear in *m* for a fixed sampler and batching scheme, *m* should be treated as a validation-tuned budget. Practical speedups may come from batched inference, caching or pre-generation when appropriate, and accelerated diffusion samplers such as DPM-Solver and DPM-Solver++ (Lu et al., 2022, 2025). One-step generation methods such as MeanFlow (Geng et al., 2025) are also promising, but any faster sampler should be validated for the target application because reducing samples or sampling steps can change generator fidelity and the final GDP decision.

Future research can further improve GDP’s computational efficiency and extend its theory beyond diffusion models. In particular, it would be valuable to develop statistical guarantees for GDP when combined with normalizing flows, large language models, or other task-adapted generators, and to study how sampler choice affects the accuracy–efficiency trade-off in practical multimodal applications.

## Figures and Tables

**Fig. 5:**
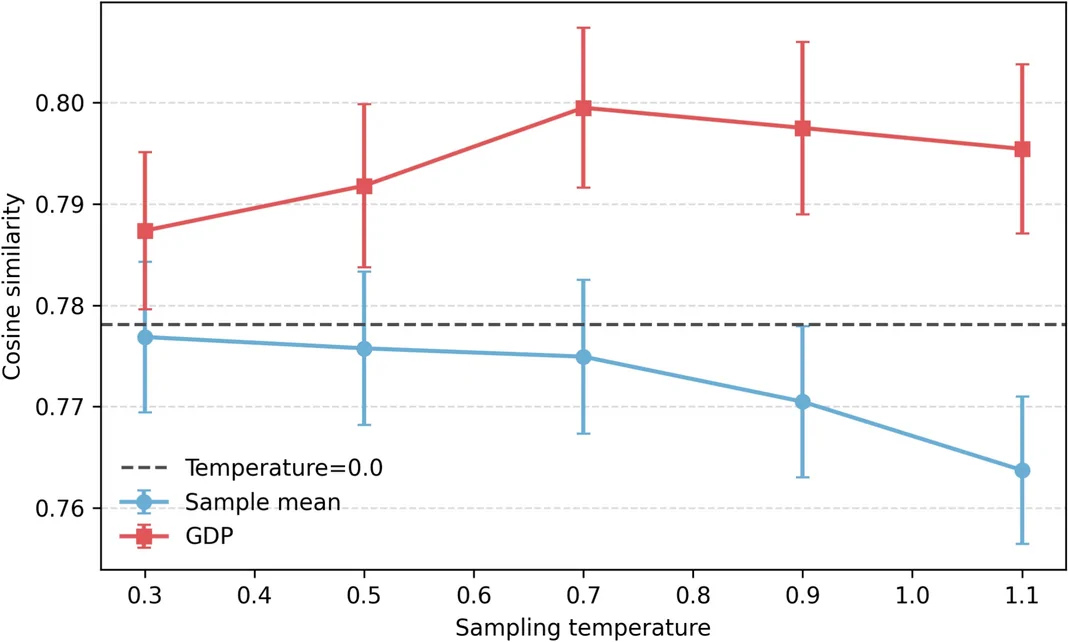
Temperature-sensitivity ablation for the Q&A experiment. For each temperature, LLaMA−3.1-8B-Instruct generates *m=50* answers for each of 100 WikiQA questions. The sample mean reports the average semantic similarity of the generated answers to the reference answer, while GDP reports the similarity of the selected answer under the semantic-consensus rule. Error bars denote standard errors across questions, and the dashed line gives the deterministic temperature 0.0 baseline

## Data Availability

No datasets were generated or analysed during the current study.

## Appendix A

### Proofs of Theorem 1

The proof proceeds by leveraging empirical process theory to derive a risk bound while simultaneously accounting for generation error. We outline the argument in four steps.

**Step 1 (Bounding the risk difference).** Let $\tilde{Y}_t$ denote the synthetic sample from $\hat{P}_{\boldsymbol{y}_t|\boldsymbol{x}_t}$ and define the corresponding excessive risk based on $\tilde{Y}_t$ as $\tilde{R}(\boldsymbol{\theta }_0,\hat{\boldsymbol{\theta }})$. By Assumption 1 and the duality theorem of Kantorovich & Rubinstein (1958), the following bounds hold:

$$\begin{aligned}&\big |R(\boldsymbol{\theta }_0,\boldsymbol{\theta })-\tilde{R}(\boldsymbol{\theta }_0,\boldsymbol{\theta })\big | \le \beta W(\hat{P}_{\boldsymbol{y}_t|\boldsymbol{x}_t},P_{\boldsymbol{y}_t|\boldsymbol{x}_t}), \\&\big |\operatorname {Var}_{\boldsymbol{y}_t|\boldsymbol{x}_t}(\ell (\boldsymbol{\theta }, \tilde{Y}_t) - \ell (\boldsymbol{\theta }_0, \tilde{Y}_t)) - \operatorname {Var}_{\boldsymbol{y}_t|\boldsymbol{x}_t}(\ell (\boldsymbol{\theta }, Y_t) - \ell (\boldsymbol{\theta }_0, Y_t)) \big | \\&\le 4c_l\beta W(\hat{P}_{\boldsymbol{y}_t|\boldsymbol{x}_t},P_{\boldsymbol{y}_t|\boldsymbol{x}_t}). \end{aligned}$$

**Step 2 (Controlling the conditional variance).** By Assumption 2,

$$\begin{aligned} \operatorname {Var}_{\tilde{\boldsymbol{y}}_t|\boldsymbol{x}_t}(\ell (\boldsymbol{\theta }, \tilde{\boldsymbol{Y}}_t) - \ell (\boldsymbol{\theta }_0, \tilde{\boldsymbol{Y}}_t))&\le \operatorname {Var}_{\boldsymbol{y}_t|\boldsymbol{x}_t}(\ell (\boldsymbol{\theta }, Y_t) - \ell (\boldsymbol{\theta }_0, Y_t)) + 4c_{\ell }\beta W, \\&\le c_v R(\boldsymbol{\theta }_0,\boldsymbol{\theta }) + 4c_{\ell }\beta W, \\&\le c_v \tilde{R}(\boldsymbol{\theta }_0,\boldsymbol{\theta }) + 2c_{\ell }(c_v + 2\beta ) W. \end{aligned}$$

where $$W=W\!\bigl (\hat{P}_{Y_t\mid X_t},\,P_{Y_t\mid X_t}\bigr )$$.

**Step 3 (Applying concentration inequality).** Setting $c_e = 1 + \beta$ yields that $\{R(\boldsymbol{\theta }_0,\boldsymbol{\theta }) \ge \varepsilon _m + c_e W\} \subset \{\tilde{R}(\boldsymbol{\theta }_0,\boldsymbol{\theta }) \ge \varepsilon _m + W\}$. Hence,

A1 $$\begin{aligned}&P_{D_{\boldsymbol{x}_t}}\big (R(\boldsymbol{\theta }_0,\boldsymbol{\theta }) \ge \varepsilon _m + c_e W\big ) \nonumber \\&\le P_{D_{\boldsymbol{x}_t}}\big (\tilde{R}(\boldsymbol{\theta }_0,\boldsymbol{\theta }) \ge \varepsilon _m + W\big ) \nonumber \\&\le \sum _{j=0}^{\infty } P_{D_{\boldsymbol{x}_t}}\big (\sup _{A_j} E_m(\tilde{L}_m(\boldsymbol{\theta }) - \tilde{L}_m(\boldsymbol{\theta }_0)) > 2^j (\varepsilon _m + W)\big ) \nonumber \\&=\sum _{j=0}^{\infty } I_j, \end{aligned}$$

where $$\tilde{L}_m(\boldsymbol{\theta }) = m^{-1} \sum _{k=1}^m \ell (\boldsymbol{\theta },\tilde{Y}^k_t; \boldsymbol{x}_t)$$ and $$A_j = \{\boldsymbol{\theta }: 2^j(\varepsilon _m+W) < \tilde{R}(\boldsymbol{\theta }_0,\boldsymbol{\theta }) \le 2^{j+1}(\varepsilon _m+W)\}$$.

Next, we bound each term $I_j$ (*j=0,⋯ ,*) in (A1) by Lemma 1 separately. Toward this end, we verify (A2) and (A3). Note that

$$\begin{aligned} \phi (2^j(W+\varepsilon _m),m)\ge&\frac{m (2^j(\varepsilon _m+W))^{2}}{2[4(c_v2^{j+1}+2c_{\ell }c_v+4c_{\ell }\lambda )(\varepsilon _m+W)+2^j(\varepsilon _m+W) \frac{c_{\ell }}{3} ]}\\&\ge \frac{m 2^j(\varepsilon _m+W)}{c_k}, \end{aligned}$$

where $c_k=2[8(c_{v}+c_{v}c_{\ell }+2c_{\ell }\lambda )+\frac{c_{\ell }}{3}]$. This implies (A2). Moreover, $\varepsilon _m$ is required to satisfy the entropy inequality,

$$ \int _{r\varepsilon _m^2/8}^{4 c^{1/2}_{v} \varepsilon _m} H^{1/2}(u, \mathcal {L}) \, du \le \frac{r^{3/2}}{2^{10}} m^{1/2} \varepsilon _m^2. $$

where $\mathcal {L}=\{l(\boldsymbol{\theta },\boldsymbol{y}),\boldsymbol{\theta }\in [-c_b,c_b]^{d_{\boldsymbol{\theta }}}\}$. Then, $H(u, \mathcal {L})\le \frac{1}{d_{\boldsymbol{\theta }}}\log (\frac{2B\beta }{u})$. This leads to $\varepsilon _m>c_{\varepsilon }m^{-\frac{1}{2}}\log m$, for some $c_{\varepsilon }$ depending on $c_b$, $c_v$, *r* and $d_{\boldsymbol{\theta }}$. Specifically, $c_{\varepsilon }$ can be set as $2^{14}\frac{c_v^{1/2}}{d_{\boldsymbol{\theta }}}$ when *m* is large enough. Then, (A3) holds. Hence, by Lemma 1, we obtain that

$$\begin{aligned} P_{D_{\boldsymbol{x}_t}}\big (R(\boldsymbol{\theta }_0,\boldsymbol{\theta }) \ge \varepsilon _m + c_e W\big ) \le 3\exp \left( - (1-r) \frac{m \varepsilon _m}{c_k} \right) . \end{aligned}$$

**Step 4 (Taking expectations).** To derive the risk bound, let $S = R(\boldsymbol{\theta }_0, \hat{\boldsymbol{\theta }}) - c_e W$. Then,

$$\begin{aligned} \mathbb {E}_{D_{\boldsymbol{x}_t}} \frac{S}{\varepsilon _m}&\le 1 + \int _1^{\infty } P(S \ge x\varepsilon _m)\,dx + \frac{1}{\varepsilon _m} P(S > \varepsilon _m) \le 2. \end{aligned}$$

Hence,

$$\begin{aligned} \mathbb {E}_{D_{\boldsymbol{x}_t}} R(\boldsymbol{\theta }_0, \hat{\boldsymbol{\theta }}) \le c_e W + 2c_{\varepsilon } m^{-1/2} \log m. \end{aligned}$$

By the assumption $\mathbb {E} W(\hat{P}_{\boldsymbol{y}_t|\boldsymbol{x}_t}, P_{\boldsymbol{y}_t|\boldsymbol{x}_t}) \le \gamma _n$, taking expectations on both sides completes the proof. *□ *

### Lemma 1

Assume that $f(\boldsymbol{Z}_j) \in \mathcal F$ satisfies the Bernstein condition with some constant *U* for an independent sample $\boldsymbol{Z}_1,\cdots ,\boldsymbol{Z}_m$, $\mathbb {E}|f(\boldsymbol{Z}_j)-\mathbb {E}f(\boldsymbol{Z}_j)|^k\le \frac{1}{2}k!v_j U^{k-2}$; *j=1,… ,m*; *k≥ 2*. Let $\varphi (\varepsilon _m,v,\mathcal{F})=\frac{m\varepsilon _m^{2}}{2[4v^2+ \varepsilon _m U /3 ]}$, where $\frac{1}{m}\sum _{j=1}^m \textrm{Var}(f(\boldsymbol{Z}_j))\le m^{-1} \sum _{j=1}^m v_j\le v$. Assume that

A2 $$\begin{aligned} \varepsilon _m \le r v/4U, \end{aligned}$$

with *0<r<1* and

A3 $$\begin{aligned} \int _{r \varepsilon _m/8}^{\sqrt{v}} H^{1/2}(u,\mathcal{F}) du \le \sqrt{n}\varepsilon _m r^{3/2}/2^{10}, \end{aligned}$$

where $H(\cdot ,\mathcal{F})$ is the bracketing $L_2$ metric entropy of the function class $\mathcal{F}$, then

$$\begin{aligned}&P^{*}(\sup _{\{f \in \mathcal{F}\}} n^{-1} \sum _{i=1}^n (f(\boldsymbol{Y}^i) -\operatorname {E}f(\boldsymbol{Y}^i)) \ge \varepsilon _m) \\&\le 3 \exp (-(1-r) \varphi (\varepsilon _m,v,m)), \end{aligned}$$

where $P^{*}$ denotes the outer probability measure for $\boldsymbol{Z}_1,\cdots ,\boldsymbol{Z}_m$.

## Appendix B Experiment Details

### Experiment Settings for Sect. 4.1

For quantile regression, we employ XGBoost with the quantile loss objective. Hyperparameter tuning is conducted using GridSearchCV with 3-fold cross-validation. The search space includes the following parameters: learning rate (0.01, 0.04, 0.1), maximum tree depth (3, 5, 7), and number of estimators (50, 100, 150). The best hyperparameters are selected based on the pinball loss metric. Once tuning is complete, the optimal model is used to generate predictions on the test set.

For the DQR model, we utilize a ReLU network, with 3-fold cross-validation to determine the optimal network width (64, 128) and network depth (3, 5, 10). During training, the Adam optimizer is used with a fixed learning rate of $1 \times 10^{-4}$ for a maximum of 200 epochs. The model is trained with a batch size of 512, and early stopping is applied using the validation set, with a maximum patience of 20 epochs.

For the GDP method, the neural network architecture for the conditional diffusion models consists of two components: a ReLU network for condition embedding and a ReLU network for score matching, both set to the same network width. Additionally, we employ a sinusoidal time embedding to project the timestep into a vector. The diffusion model operates over 1000 timesteps, ranging from $1 \times 10^{-4}$ to 0.02. Hyperparameter tuning is performed using 3-fold cross-validation, optimizing the condition embedding dimension (64, 128), time embedding dimension (16, 32, 64), network width (64, 128, 256), and network depth (3, 5). The training strategy follows the same procedure as the DQR model.

### Experiment Settings for Sect. 4.2

For the GDP method, the neural network architecture for the conditional diffusion models consists of two components: a ReLU network for condition embedding and a ReLU network for score matching, both set to the same network width. Additionally, we employ a sinusoidal time embedding to project the timestep into a vector. The condition-embedding dimension is 256, the time-embedding dimension is 64, and the score network has width 256 and depth 4. Training (optimizer, learning rate, batch size, and number of epochs) follows the same used for quantile regression experiments.

For the kernel method, We adopt the kernel-based modal regression implementation from https://github.com/yenchic/ModalRegression. The bandwidths are selected by leave-one-out cross-validation (LOOCV) on the joint kernel density.

### Experiment Settings for Sect. 4.3

All experiments were conducted on a compute node equipped with two Intel Xeon Gold 6126 CPUs (*2× 12* cores, 48 threads total, 2.60 GHz base frequency) and an NVIDIA Tesla V100 PCIe GPU (32 GB HBM2, NVIDIA driver 550.163.01, CUDA 12.4).

**Dataset:** In the Yelp review experiment, we use the review text as input and the star ratings $\{1,2,3,4,5\}$ as the target to train our prediction model. For the UTKFace dataset, we combine tabular features (gender and ethnicity) with the facial image to predict each subject’s age. In the Shopee dataset, we select four categories—Babypants, BabyShirt, WomenCasualShoes, and WomenChiffonTop—and use their corresponding images to train and evaluate the models.

**Multimodal predictor:** All models in this section are implemented using AutoGluon’s “multimodal” preset. We include CatBoost, XGBoost, a feed-forward neural network, and the built-in multimodal predictor. The text backbone is “google/electra-small-discriminator” and the vision backbone is “ResNet-18”. During training, we hold out 20% of the data for validation (holdout_frac=0.2).

**TTT and TIP baselines:** For Yelp, we adapt the official TTT implementation from the public GitHub repository https://github.com/thomas-bonnier/TabularTextTransformer to the same split protocol as the main Yelp experiment. Specifically, we define the target domain by cool*≠ 0*, split it into 80% training and 20% testing sets, reserve 20% of the target training set for validation, and train the TTT baseline on the union of the source reviews and the remaining target training reviews. The adapted TTT model uses the review text together with a categorical cool_bin domain indicator and a standardized numerical cool feature. For UTKFace, we adapt the official TIP implementation from the public GitHub repository https://github.com/siyi-wind/TIP to the age-regression setting. The resulting TIP baseline combines a pretrained image encoder with tabular gender and ethnicity inputs through the official multimodal architecture, with the downstream head modified from classification to scalar regression. Both baselines are evaluated on the same five random train/test splits as Table 3.

**Probabilistic baselines:** NGBoost and BNN serve as distribution-aware baselines. NGBoost is fit with the ngboost package (Duan et al., 2020), while BNN is implemented using IntelLabs’ Bayesian-Torch package (Krishnan et al., 2022) by converting feed-forward prediction heads into variational Bayesian layers. These baselines are trained on fixed representations rather than end-to-end from raw modalities: Yelp uses TF-IDF text features together with the cool metadata, UTKFace uses ResNet-18 image embeddings concatenated with gender and ethnicity indicators, and Shopee-IET uses the saved AutoMM image embeddings from each split. The runtime reported for NGBoost and BNN starts after feature construction, so it excludes training the upstream feature extractor and the embedding extraction pass.

**GDP-diffusion:** For the GDP-diffusion method, we train a conditional diffusion model using the same backbone embeddings (“electra-small-discriminator” and “ResNet-18”). The architecture is as follows:

- **Input:** A noisy scalar value, conditional inputs of the text representation from the text model and image representation from the image model, and time step $\tau \in [1, T]$.
- **Condition encoding:** A multi-layer perceptron encodes the condition *x* via $h_{\text {cond}} = f_{\text {cond}}(x) = \text {MLP}(x) \in \mathbb {R}^{h},$ where *h* is the hidden dimension.
- **Time embedding:** The timestep *t* is embedded using a learned transformation: $h_{\tau } = f_{\text {time}}(\tau ) = \text {MLP}(\tau ) \in \mathbb {R}^{e},$ where *e* is the time embedding dimension.
- **Diffusion backbone:** The final input is the concatenation of $(x, h_{\text {cond}}, h_t)$, followed by residual blocks: $h = \text {ReLU}(W_1[x, h_{\text {cond}}, h_{\tau }] + b_1), \quad h = \text {ResBlock}^{(L)}(h).$
- **Output:** The model predicts the noise $\hat{\epsilon }$ using: $\hat{\epsilon } = W_{\text {out}} h + b_{\text {out}}$ (Table 5).

**Table 5:** Hyperparameter search space for diffusion model training

Hyperparameter	Search space
hidden_dim	{256, 512, 1024}
time_embed_dim	{64, 128, 256}
layers	Integer in [3, 10]
dropout_p	Float in [0.0, 0.05]
learning_rate	Log-uniform in $[10^{-5}, 10^{-4}]$
noise_steps	Integer in [300, 1000]
Timestep end	Float in [0.01, 0.02]
batch size	{32, 64, 128}

We set aside 20% of the training data as a validation set. Each configuration is trained on the training set and evaluated on the validation set using the mean squared error between predicted and true noise vectors. The best-performing model under this objective is selected for GDP prediction and evaluation.

For the GDP-tr variant, we first train on the source data, then freeze the text backbone and fine-tune the diffusion model on the target data using the same procedure.

For each dataset, we generate *m=100* synthetic samples and report the prediction as the mode for classification tasks and the mean for regression tasks.

### Experiment Settings for Sect. 4.4

In the image-captioning experiment, we employ a conditional diffusion model to encode multimodal data, including images and text data into a shared latent embedding space for caption generation, as described in Sect. 3.2.

Our approach deviates from standard implementations in several key ways: (1) it uniquely integrates CLIP and SentenceBERT for multimodal alignment, (2) it employs a residual network for reverse diffusion instead of the more commonly used U-Net Ronneberger et al. (2015), and (3) it utilizes vec2text to decode denoised embeddings into captions. While building on existing principles, this method makes it a distinctive contribution to multimodal text generation.

To represent image-caption pairs in this unified space, we use SentenceBERT and CLIP: SentenceBERT (GTR-T5-Base, https://huggingface.co/sentence-transformers/gtr-t5-base) captures rich linguistic semantics from textual descriptions, while CLIP (CLIP-ViT-B-32, https://huggingface.co/sentence-transformers/clip-ViT-B-32) extracts aligned image and text embeddings. All embeddings are standardized to a common dimensionality using trainable projection layers for alignment and then fused through concatenation. By combining SentenceBERT and CLIP, this approach enhances caption quality by refining textual representations beyond what CLIP alone can achieve.

Building on these joint embeddings, we apply a Gaussian diffusion process for conditional caption generation, iteratively injecting and refining noise in the text embeddings based on the given image. We model the reverse diffusion process by a residual network, progressively removing noise while conditioning the image, ensuring the generated caption remains visually relevant. Specifically, the diffusion model inputs a noisy text embedding from GTR-T5-Base, a condition embedding from CLIP-ViT-B-32, and a timestep indicator. These inputs are projected into a unified hidden space of 2048 dimensions, with the timestep further encoded into a time embedding through a two-layer MLP, also with a 2048-dimensional hidden layer.

At the core of the reverse diffusion process is a stack of ten residual blocks, each performing a linear transformation, followed by layer normalization and a ReLU activation to ensure stable training and efficient information flow. After iterative refinement through these blocks, a final projection layer maps the processed hidden representation back to the text embedding space. This mapping allows the model to predict the denoised text embedding at each diffusion step, which is then decoded by vec2text (https://huggingface.co/ielabgroup/vec2text_gtr-base-st_corrector) to generate the final image caption.

In the training process, the model is trained for 1000 epochs with Adam optimizer with a fixed learning rate of $1 \times 10^{-4}$ and batch size 256.

The BLIP image-captioning model (https://huggingface.co/Salesforce/blip-image-captioning-base) is implemented with a temperature setting of 1.0.

### Experiment Settings for Sect. 4.5

For the Q&A experiment, LLaMA−3.1-8B-Instruct generates one deterministic answer with temperature 0.0 and *m=50* sampled answers with temperature 0.7 for each question. Semantic evaluation is based on cosine similarity between answer embeddings and the reference-answer embedding, using Google’s text-embedding-004. GDP selects the candidate answer whose embedding has the largest average cosine similarity to the other *m=50* sampled-answer embeddings.

In Table 5, we further conduct a temperature-sensitivity ablation for the Q&A experiment using the same 100 WikiQA test questions. For each temperature in $\{0.3,0.5,0.7,0.9,1.1\}$, LLaMA−3.1-8B-Instruct generates *m=50* sampled answers per question, and GDP applies the same semantic-consensus selection rule as in Sect. 4.5. As shown in Fig. 5, GDP consistently improves over the unselected sample mean across all temperatures. The best performance is attained near the default temperature 0.7, while very high temperatures reduce the average quality of unselected samples because the generated answers become more variable. This ablation supports the use of a moderate sampling temperature to balance candidate diversity and answer quality.

For the LLM-Judge baseline in Fig. 4, following the LLM-as-a-judge paradigm of Zheng et al. (2023), we use Gemini Flash through Vertex AI with temperature 0. The judge is given only the question and the same *m=50* generated candidate answers; the reference answer is not included in the judge prompt and is used only for final evaluation. Candidate answers are indexed from 0 to 49. The prompt template is: You are an expert verifier for an open-domain question-answering task. Select the single candidate answer that best answers the question. Prefer answers that are factually correct, directly responsive, and not padded with irrelevant text. Return only the 0-based integer index of the best candidate. Do not return JSON, Markdown, explanation, or any other text. Question: $\{\text {question}\}$ Candidate answers: $[0]\ \{\text {candidate answer 0}\}, \ldots , [49]\ \{\text {candidate answer 49}\}$ Return exactly one integer from 0 to 49. The returned index identifies the LLM-Judge-selected answer, which is then evaluated using the same reference-answer embedding similarity as the other methods.

## Appendix C Diffusion Models and Generation Accuracy Theory

### Diffusion Modeling

Recent advancements in generative modeling provide compelling solutions to challenges in supervised learning by harnessing their robust data synthesis capabilities. In particular, models such as diffusion models and normalizing flows—each tailored to specific domains—enable the creation of high-quality synthetic data that closely approximates the true underlying data-generating distribution.

In this section, we rigorously derive the concept of diffusion generalization accuracy. In conjunction with Theorem 1 presented in Sect. 2.2, our derivation establishes a statistical guarantee for the performance of diffusion-GDP in the context of diffusion modeling. Furthermore, the multimodal diffusion model discussed in Sect. 3.2 builds upon this framework by integrating diverse data modalities. Then, we employ an encoder-decoder architecture to generate vector embeddings capturing the intrinsic properties of each modality, followed by diffusion modeling on vector embeddings.

We begin our discussion with a Gaussian diffusion model.

**Forward process. ** The forward process systematically transforms a random vector $\boldsymbol{U}(0)$ into white noise by progressively injecting white noise into a differential equation defined with the Ornstein-Uhlenbeck process, leading to diffused distributions from the initial state $\boldsymbol{U}(0)$:

C4 $$\begin{aligned} \textrm{d}\boldsymbol{U}(\tau )=-{b}_{\tau } \boldsymbol{U}(\tau )\textrm{d}\tau +\sqrt{2{b}_\tau }\textrm{d}W(\tau ),\quad \tau \ge 0, \end{aligned}$$

where $\boldsymbol{U}(\tau )$ has a probability density $p_{\boldsymbol{u}(\tau )}$, $\{W(\tau )\}_{\tau \ge 0}$ represents a standard Wiener process and ${b}_t$ is a non-decreasing weight function. Under (C4), $\boldsymbol{U}(\tau )$ given $\boldsymbol{U}(0)$ follows $N(\mu _{\tau }\boldsymbol{U}(0),\sigma ^2_{\tau }\boldsymbol{I})$, where $\mu _{\tau }=\exp (-\int _0^\tau {b}_s\textrm{d} s)$ and $\sigma ^2_{\tau }=1-\mu _{\tau }^2$. Here, we set ${b}_s=1$, which results in $\mu _{\tau }=\exp {(-\tau )}$ and $\sigma ^2_{\tau }=1-\exp {(-2\tau )}$. Practically, the process terminates at a sufficiently large $\overline{\tau }$, ensuring the distribution of $\boldsymbol{U}(\tau )$, a mixture of $\boldsymbol{U}(0)$ and white noise, resembles the standard Gaussian vector.

**Backward process.** Given $\boldsymbol{U}(\overline{\tau })$ in (C4), a backward process is employed for sample generation for $\boldsymbol{U}(0)$. Assuming (C4) satisfies certain conditions Anderson (1982), the backward process $\boldsymbol{V}(\tau )=\boldsymbol{U}(\overline{\tau }-\tau )$, starting with $\boldsymbol{U}(\overline{\tau })$, is derived as:

C5 $$\begin{aligned} \textrm{d}\boldsymbol{V}(\tau )=&{b}_{\overline{\tau }-\tau }(\boldsymbol{V}(\tau )+2\nabla \log p_ {\boldsymbol{u}(\overline{\tau }-\tau )}(\boldsymbol{U}(\overline{\tau }-\tau ))\textrm{d}\tau +\sqrt{2{b}_{\overline{\tau }-\tau }}\textrm{d}W(\tau ), \end{aligned}$$

where $\nabla \log p_{\boldsymbol{x}}$ is the score function which represents the gradient of $\log p_{\boldsymbol{x}}$.

**Score matching.** To estimate the unknown score function, we minimize a matching loss between the score and its approximator *ψ *: $\int _{0}^{\overline{\tau }}\textrm{E}_{\boldsymbol{u}(\tau )}\Vert \nabla \log p_{\boldsymbol{u}(\tau )}(\boldsymbol{U}(\tau ))-\psi (\boldsymbol{U}(\tau ),\tau )\Vert ^2\textrm{d}\tau$, where $\Vert \boldsymbol{u}\Vert =\sqrt{\sum ^{d_u}_{j=1}\boldsymbol{u}_j^2}$ is the Euclidean norm, which is equivalent to minimizing the following loss (Oko et al., 2023),

C6 $$\begin{aligned} \int _{\underline{\tau }}^{\overline{\tau }}\textrm{E}_{\boldsymbol{u}(0)}\textrm{E}_{\boldsymbol{u}(\tau )|\boldsymbol{u}(0)}\Vert \nabla \log p_{\boldsymbol{u}(\tau )|\boldsymbol{u}(0)}(\boldsymbol{U}(\tau )|\boldsymbol{U}(0))-\theta (\boldsymbol{U}(\tau ),\tau )\Vert ^2\textrm{d}\tau , \end{aligned}$$

with $\underline{\tau }=0$. In practice, to avoid score explosion due to $\nabla \log p_{\boldsymbol{x}(\tau )|\boldsymbol{x}(0)} \rightarrow \infty$ as $\tau \rightarrow 0$ and to ensure training stability, we restrict the integral interval to $\underline{\tau }>0$ (Chen et al., 2023; Oko et al., 2023) in the loss function. Then, both the integral and $\textrm{E}_{\boldsymbol{u}(\tau )|\boldsymbol{u}(0)}$ can be precisely approximated by sampling *τ * from a uniform distribution on $[\underline{\tau },\overline{\tau }]$ and a sample of $\boldsymbol{U}(0)$ from the conditional distribution of $\boldsymbol{U}(\tau )$ given $\boldsymbol{U}(0)$.

**Generation.** To generate a random sample of $\boldsymbol{V}(\tau )$, we replace the score $\nabla \log p_{\boldsymbol{u}(\overline{\tau }-\tau )}$ by its estimate $\hat{\psi }$ in (C5) to yield $\boldsymbol{V}(\tau )$ in the backward equation. For implementation, we may utilize a discrete-time approximation of the sampling process, facilitated by numerical methods for solving stochastic differential equations, such as Euler-Maruyama and stochastic Runge–Kutta methods (Song et al., 2020).

**Neural network. ** An $\mathbb {L}$-layer network *Φ * is defined by a composite function $\Phi (\boldsymbol{x})=(\boldsymbol{\textrm{A}}_\mathbb {L}\sigma (\cdot )+\boldsymbol{b}_\mathbb {L})\circ \cdots (\boldsymbol{\textrm{A}}_2\sigma (\cdot )+\boldsymbol{b}_2)\circ (\boldsymbol{\textrm{A}}_1\boldsymbol{x}+\boldsymbol{b}_1),$ where $\boldsymbol{\textrm{A}}_i\in \mathbb R^{d_{i+1}\times d_i}$ is a weight matrix and $\boldsymbol{b}_i \in \mathbb R^{d_{i+1}}$ is the bias of a linear transformation of the *i*-th layer, and *σ * is the ReLU activation function, defined as $\sigma (\boldsymbol{x})=\max (\boldsymbol{x},0)$. Then, the parameter space *Ψ * is set as $\textrm{NN}(\mathbb {L},\mathbb {W},\mathbb {S},\mathbb {B},\mathbb {E})$ with $\mathbb {L}$ layers, a maximum width of $\mathbb {W}$, effective parameter number $\mathbb {S}$, the sup-norm $\mathbb {B}$, and parameter bound $\mathbb {E}$:

C7 $$\begin{aligned} \textrm{NN}(d_{in},d_{out},\mathbb {L},\mathbb {W},\mathbb {S},\mathbb {B},\mathbb {E}) =&\{ \Phi : d_1=d_{in}, d_{\mathbb {L}+1}=d_{out}, \max _{1\le i\le \mathbb {L}}d_i\le \mathbb {W}, \nonumber \\&\sum _{i=1}^{\mathbb {L}}(\Vert \boldsymbol{\textrm{A}}_i\Vert _0+ \Vert \boldsymbol{b}_i\Vert _0)\le \mathbb {S}, \Vert \Phi \Vert _{\infty }\le \mathbb {B}, \nonumber \\&\max _{1\le i\le \mathbb {L}}(\Vert \boldsymbol{\textrm{A}}_i\Vert _{\infty }, \Vert \boldsymbol{b}_i\Vert _{\infty })\le \mathbb {E} \}, \end{aligned}$$

where $\Vert \cdot \Vert _{\infty }$ is the maximal magnitude of entries and $\Vert \cdot \Vert _0$ is the number of nonzero entries.

To learn the latent distribution of $\boldsymbol{U}$ given $\boldsymbol{X}$, we use the conditional diffusion model which leads to the following conditional score matching loss,

$$\begin{aligned} l_{t}(\boldsymbol{u},h(\boldsymbol{x});\psi )= \int _{\underline{\tau }}^{\overline{\tau }}\textrm{E}_{\boldsymbol{u}(\tau )|\boldsymbol{u}(0)}\Vert \nabla \log p_{\boldsymbol{u}(\tau )|\boldsymbol{u}(0)}(\boldsymbol{U}(\tau )|\boldsymbol{u})-\psi (\boldsymbol{U}(\tau ),h(\boldsymbol{x}),\tau )\Vert ^2\textrm{d}\tau . \end{aligned}$$

Before proceeding, we first introduce the definition of smooth class. Let $\boldsymbol{\alpha }$ be multi-index with $|\boldsymbol{\alpha }| \le \lfloor r\rfloor$, where $\lfloor r\rfloor$ is the integer part of *r>0*. A Hölder ball $\mathcal {C}^{r}(\mathcal {D},\mathbb R^m,B)$ of radius *B* with the degree of smoothness *r* from domain $\mathcal {D}$ to $\mathbb R^m$ is defined by:

$$\begin{aligned} \big \{(g_1,\cdots ,g_m): \max _{1\le l\le m}\big (&\max _{|\boldsymbol{\alpha }| \le \lfloor r\rfloor }\sup _{\begin{array}{c} \boldsymbol{x} \end{array}} |\partial ^{\boldsymbol{\alpha }} g_l(\boldsymbol{x})| + \max _{|\boldsymbol{\alpha }|=\lfloor r\rfloor }\sup _{\begin{array}{c} \boldsymbol{x} \ne \boldsymbol{y} \end{array}}\frac{|\partial ^{\boldsymbol{\alpha }} g_l(\boldsymbol{x}) - \partial ^{\boldsymbol{\alpha }} g_l(\boldsymbol{y})|}{\Vert \boldsymbol{x} - \boldsymbol{y}\Vert ^{r-\lfloor r\rfloor }}\big ) < B \big \}. \end{aligned}$$

### Theory

In our transfer learning scenarios, we consider a *source task* and a *target task*. The source task (indexed by *s*) is typically associated with a large amount of labeled data and well-trained models, while the target task (indexed by *t*) may have limited labeled data. In this framework, we leverage a pretrained encoder-decoder pair obtained from the source task to process data in the target task. In particular, the encoder $\hat{f}$ maps the multimodal inputs into a latent embedding space, and the decoder $\hat{g}$ reconstructs the original data from the latent space. Within the latent space, a Gaussian diffusion model is applied to refine or generate new embeddings. Finally, the decoder $\hat{g}$ maps these refined embeddings back to the original multimodal space. This approach facilitates efficient processing and generation of complex multimodal data while benefiting from the information learned in the source task.

Let $(\hat{f},\hat{g})$ denote a pretrained encoder-decoder pair. For each task $j \in \{s,t\}$, define the latent representation as $\boldsymbol{U}_j = \hat{f}(\boldsymbol{Y})$, and the corresponding reconstruction by $\boldsymbol{Y}_j = \hat{g}(\boldsymbol{U}_j)$. Here, $\boldsymbol{Y}_j$ denotes the response variable, respectively, for task *j*. The source task benefits from a large amount of data and has lower reconstruction error, while the target task may experience a higher error due to domain differences or fewer training samples.

#### Assumption 3

*(Reconstruction error)* Suppose that the pretrained encoder-decoder $(\hat{f},\hat{g})$ satisfies

$$ \operatorname {E}\Bigl [\Vert \hat{g}(\hat{f}(\boldsymbol{y}_t))-\boldsymbol{y}_t\Vert \Bigr ] \le \varepsilon _s, $$

where $l_{r,t}$ is an appropriate reconstruction loss function. Here, the error $\varepsilon _s$ is related to the source task performance and is assumed to be small due to abundant source data and thorough pretraining. Furthermore, assume that the decoder $\hat{g}$ is Lipschitz continuous with Lipschitz constant $\lambda _g$, i.e., for any $\boldsymbol{u}, \boldsymbol{u}'$,

$$ \Vert \hat{g}(\boldsymbol{u}) - \hat{g}(\boldsymbol{u}')\Vert \le \lambda _g \Vert \boldsymbol{u} - \boldsymbol{u}'\Vert . $$

#### Assumption 4

*(Latent density)* Assume that the conditional density of the latent representation $\boldsymbol{U}_t = \hat{f}(\boldsymbol{Y}_t)$ given a feature extraction $\hat{h}(\boldsymbol{x}_t)$ (where $\hat{h}$ is a known function on $\boldsymbol{X}_t$) $p^0_{\boldsymbol{u}_t|\boldsymbol{x}_t}(\boldsymbol{u}_t|\boldsymbol{x}_t)$ can be written as

$$ p^0_{\boldsymbol{u}_t|\boldsymbol{x}_t}(\boldsymbol{u}_t|\boldsymbol{x}_t) = \exp \Bigl (-\frac{c_1}{2}\Vert \boldsymbol{u}_t\Vert ^2\Bigr ) \cdot k_t\bigl (\boldsymbol{u}_t,\hat{h}(\boldsymbol{x}_t)\bigr ), $$

where $c_1>0$ is a constant, and $k_t: \mathbb R^{d_u} \times [0,1]^{d_h} \rightarrow \mathbb R^+$ is a nonnegative function that is bounded away from zero. In addition, assume that $k_t$ belongs to a Hölder ball $\mathcal {C}^{r_u}(\mathbb R^{d_u} \times [0,1]^{d_h}, \mathbb R, B_t)$ with the degree of smoothness parameter $r_u>0$ and bound $B_t>0$, where $d_u$ is the dimension of $\boldsymbol{u}_t$ and $d_h$ is the dimension of $\hat{h}(\boldsymbol{x}_t)$.

#### Theorem 2

(Conditional diffusion via transfer learning) Under Assumptions 3 and 4, the expected Wasserstein distance between the true conditional distribution between the true conditional distribution $P^0_{\boldsymbol{y}_t|\boldsymbol{x}_t}$ and its synthetic counterpart $\hat{P}_{\boldsymbol{y}_t|\boldsymbol{x}_t}$ generated via transfer learning satisfies

$$ \operatorname {E}\Bigl [W\Bigl (P^0_{\boldsymbol{y}_t|\boldsymbol{x}_t}, \hat{P}_{\boldsymbol{y}_t|\boldsymbol{x}_t}\Bigr )\Bigr ] = O\Bigl (n^{-\frac{r_u}{d_u+d_h+2r_u}}\log ^{m_u} n + \varepsilon _s\Bigr ), $$

where *n* is the target task sample size, $\varepsilon _s$ denotes the reconstruction error on the target task, which is influenced by the performance on the source task, and $m_u = \max \Bigl \{\frac{19}{2},\frac{r_u}{2}+1\Bigr \}$.

Typically, $\varepsilon _s \ll n^{-\frac{r_u}{d_u+d_h+2r_u}}\log ^{m_u} n$ is negligible, as it is mitigated by leveraging a large pre-trained dataset.

#### of Theorem 2

Let $\bar{P}_{\boldsymbol{y}_t|\boldsymbol{x}_t}$ denote the distribution of the reconstructed target variable $\bar{\boldsymbol{Y}}_t = \hat{g}(\boldsymbol{U}_t)$, where $\boldsymbol{U}_t = \hat{f}(\boldsymbol{Y}_t)$ is the latent representation.

**Step 1 (Error decomposition).** We first decompose the Wasserstein distance as follows:

$$ W\Bigl (P^0_{\boldsymbol{y}_t|\boldsymbol{x}_t}, \hat{P}_{\boldsymbol{y}_t|\boldsymbol{x}_t}\Bigr ) \le W\Bigl (\bar{P}_{\boldsymbol{y}_t|\boldsymbol{x}_t}, \hat{P}_{\boldsymbol{y}_t|\boldsymbol{x}_t}\Bigr ) + W\Bigl (P^0_{\boldsymbol{y}_t|\boldsymbol{x}_t}, \bar{P}_{\boldsymbol{y}_t|\boldsymbol{x}_t}\Bigr ). $$

The first term on the right-hand side captures the error due to the diffusion model in the latent space, and the second term reflects the reconstruction error incurred by $\hat{g}$.

**Step 2 (Bounding the reconstruction error with** $\varepsilon _s$ **).** By the Kantorovich–Rubinstein duality Kantorovich & Rubinstein (1958) and using the Lipschitz continuity of $\hat{g}$, we obtain

$$ W\Bigl (\bar{P}_{\boldsymbol{y}_t|\boldsymbol{x}_t}, \hat{P}_{\boldsymbol{y}_t|\boldsymbol{x}_t}\Bigr ) \le \lambda _g \, W\Bigl (\bar{P}_{\boldsymbol{u}_t|\boldsymbol{x}_t}, \hat{P}_{\boldsymbol{u}_t|\boldsymbol{x}_t}\Bigr ), $$

where $\bar{P}_{\boldsymbol{u}_t|\boldsymbol{x}_t}$ and $\hat{P}_{\boldsymbol{u}_t|\boldsymbol{x}_t}$ denote the true and estimated distributions of the latent variable $\boldsymbol{u}_t$, respectively.

**Step 3 (Bounding the generation error term by diffusion theory).** Under Assumption 4 and by applying Theorem 2 of Tian & Shen (2024), it follows that

$$ \operatorname {E}\Bigl [W\Bigl (\bar{P}_{\boldsymbol{u}_t|\boldsymbol{x}_t}, \hat{P}_{\boldsymbol{u}_t|\boldsymbol{x}_t}\Bigr )\Bigr ] = O\Bigl (n_t^{-\frac{r_u}{d_u+d_h+2r_u}}\log ^{m_u} n_t\Bigr ). $$

For the second term, by definition of the reconstruction error, we have

$$ W\Bigl (P^0_{\boldsymbol{y}_t|\boldsymbol{x}_t}, \bar{P}_{\boldsymbol{y}_t|\boldsymbol{x}_t}\Bigr ) \le \operatorname {E}\Bigl [\Vert \hat{g}(\hat{f}(\boldsymbol{y}_t))-\boldsymbol{y}_t\Vert \Bigr ] \le \varepsilon _s. $$

Combining these bounds yields

$$ \operatorname {E}\Bigl [W\Bigl (P^0_{\boldsymbol{y}_t|\boldsymbol{x}_t}, \hat{P}_{\boldsymbol{y}_t|\boldsymbol{x}_t}\Bigr )\Bigr ] \le \lambda _g \, O\Bigl (n_t^{-\frac{r_u}{d_u+d_h+2r_u}}\log ^{m_u} n_t\Bigr ) + \varepsilon _s, $$

which establishes the claimed result. This completes the proof. *□ *

Next, we present a result for non-transfer diffusion that does not leverage the source learning.

#### Corollary 1

(Non-transfer conditional diffusion generation) Under the conditions of Theorem 2 with *f*, *g*, *h* known as identical mappings, we have the error bound for the simple case,

$$ \operatorname {E}\Bigl [W\Bigl (P^0_{\boldsymbol{y}_t|\boldsymbol{x}_t}, \hat{P}_{\boldsymbol{y}_t|\boldsymbol{x}_t}\Bigr )\Bigr ] = O\Bigl (n^{-\frac{r_u}{d_y+d_x+2r_u}}\log ^{m_u} n\Bigr ). $$

This is a direct consequence of Theorem 2 with $d_u=d_y$, $d_h=d_x$ and $\varepsilon _s=0$.

## Footnotes

1 https://www.yelp.com/dataset

2 https://susanqq.github.io/UTKFace/

3 https://automl-mm-bench.s3.amazonaws.com/vision_datasets/shopee.zip
